# A Bayesian pick-the-winner design in a randomized phase II clinical trial

**DOI:** 10.18632/oncotarget.19088

**Published:** 2017-07-07

**Authors:** Dung-Tsa Chen, Po-Yu Huang, Hui-Yi Lin, Alberto A. Chiappori, Dmitry I. Gabrilovich, Eric B. Haura, Scott J. Antonia, Jhanelle E. Gray

**Affiliations:** ^1^ Department of Biostatistics and Bioinformatics, H. Lee Moffitt Cancer Center & Research Institute, Tampa, FL, USA; ^2^ Computational Intelligence Technology Center, Industrial Technology Research Institute, Taichung, Taiwan; ^3^ Biostatistics Program, School of Public Health, Louisiana State University Health Sciences Center, New Orleans, LA, USA; ^4^ Department of Thoracic Oncology, H. Lee Moffitt Cancer Center & Research Institute, Tampa, FL, USA; ^5^ Translational Tumor Immunology, The Wistar Institute, Philadelphia, PA, USA

**Keywords:** Bayesian posterior probability, Simon two-stage design, pick the winner design

## Abstract

**Purpose:**

Many phase II clinical trials evaluate unique experimental drugs/combinations through multi-arm design to expedite the screening process (early termination of ineffective drugs) and to identify the most effective drug (pick the winner) to warrant a phase III trial. Various statistical approaches have been developed for the pick-the-winner design but have been criticized for lack of objective comparison among the drug agents.

**Methods:**

We developed a Bayesian pick-the-winner design by integrating a Bayesian posterior probability with Simon two-stage design in a randomized two-arm clinical trial. The Bayesian posterior probability, as the rule to pick the winner, is defined as probability of the response rate in one arm higher than in the other arm. The posterior probability aims to determine the winner when both arms pass the second stage of the Simon two-stage design.

**Results:**

When both arms are competitive (i.e., both passing the second stage), the Bayesian posterior probability performs better to correctly identify the winner compared with the Fisher exact test in the simulation study. In comparison to a standard two-arm randomized design, the Bayesian pick-the-winner design has a higher power to determine a clear winner. In application to two studies, the approach is able to perform statistical comparison of two treatment arms and provides a winner probability (Bayesian posterior probability) to statistically justify the winning arm.

**Conclusion:**

We developed an integrated design that utilizes Bayesian posterior probability, Simon two-stage design, and randomization into a unique setting. It gives objective comparisons between the arms to determine the winner.

## INTRODUCTION

The purpose of an early-phase II clinical trial is to determine if a new drug has sufficient anti-tumor activity for further development. This is often implemented using Simon two-stage design [1] in a single arm setting. As modern biomedical research advances, numerous potential experimental agents have and continue to be developed. Thus, many phase II clinical trials include multiple arms with experimental drugs to expedite the screening process (early termination of ineffective drugs) and to identify the most effective drug (pick the winner) to warrant a phase III trial [2-8]. Various statistical approaches have been developed for the pick-the-winner design [5, 6, 9-16]. Yao et al. and Strauss and Simon used a sequential approach to screen treatments by a series of single-arm [15, 16] or two-arm [14] trials over a time domain. These approaches have some limitations, such as requiring intensive resources and the uncertainty of prior distribution of response rate [13]. Rubinstein et al. considered a standard randomized two-arm design with a large type I and II error [9]. However, the sample size remains relatively large (most with *n* > 100) even with a 20% type I error, a 20% type II error, and a 20% difference of response rate. The most common pick-the-winner design is a ranking and selection approach by Simon et al. [11]. This approach provides an attractive feature of requiring a small sample size and has various applications in clinical trial design and execution [5, 6]. However, the winner decision is not based on formal statistical comparisons but on the highest response rate, raising an issue of a high false-positive rate of this design [17].

In this study, we propose a pick-the-winner design by integrating Bayesian posterior probability with a Simon two-stage design in phase II randomized trial to determine the winner arm.

## RESULTS

### Study design (details in method section)

**Figure 1:**
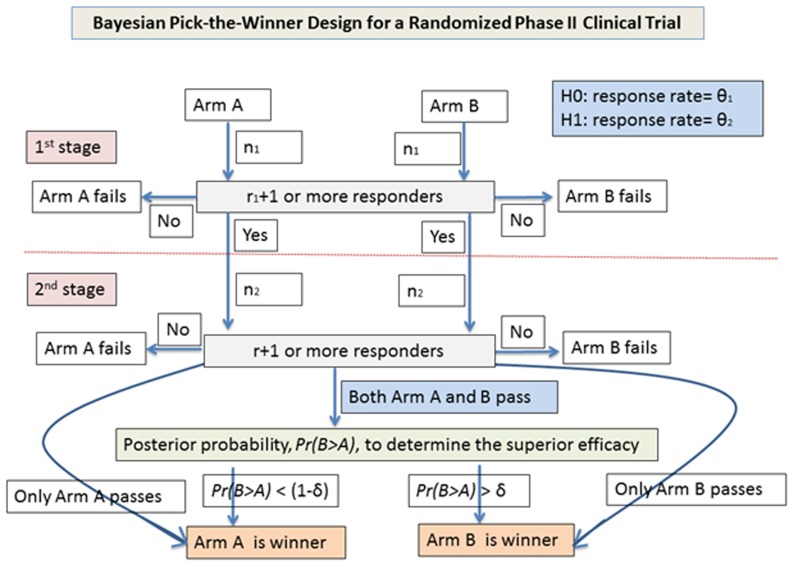
Flow chart of study design for the Bayesian pick-the-winner design in a randomized phase II trial

The design (Figure 1) considers two treatment arms, A and B, with patients randomized into one of the two treatment arm. Each treatment arm uses a Simon two-stage design [1] to evaluate the efficacy of the treatment. A treatment arm passing the second stage is considered as a “*competitive*” arm. The Bayesian posterior probability, *Pr*(*B* > *A*), defined as probability of a response rate in arm B higher than in arm A, is used to determine the winner arm when both treatment arms become competitive. With a beta prior for response rate, this probability, *Pr*(*B* > *A*), could be calculated by comparing posterior beta distributions between the two treatment arms. Arm B will be claimed as the winner if *Pr*(*B* > *A*) > δ.

### Simulation study

Simulation is used to evaluate (1) the impact of the prior distribution on *Pr*(*B* > *A*), (2) the relationship of *Pr*(*B* > *A*) and response rate difference, and (3) the effect of δ on local power and type I error. The simulation setting uses a Simon optimal two-stage design to compare 40% *versus* 20% response rate. The type I and II errors are controlled at 10%. With this design, each arm has a sample size of 17 patients in the first stage. If 4 or more patients show a response, the arm will continue to the second stage with 20 additional patients. An arm with a total number of responders greater than 10 is considered as competitive (i.e., passing the second stage). This simulation setting is labeled as “simulation setting A” for the following use.

### Effect of prior distribution

A series of prior distributions are evaluated: (i) non-informative prior, with beta(*c,c*) in both arms for *c* = 0 (Haldane prior), 0.5 (Jeffreys prior), and 1 (Bayes prior); (ii) unfavorable arm B’s prior, with beta(*c*,0) in arm A and beta(0,*c*) in arm B for *c* = 0.1, 1, and 10; (iii) favorable arm B’s prior, with beta(*0,c*) in arm A and beta(*c,0*) in arm B for *c* = 0.1, 1, and 10; and (iv) prior beta distribution based on hypothesized or observed response rate with standard deviation (SD) of 0.1 (e.g., for a comparison of 40% in arm B *versus* 20% in arm A, the prior beta distribution based on the hypothesized response rate will have a mean of 40% and 20% for arm B and A, respectively, with SD = 0.1).

With the simulation setting A and assumption of a response rate of 20% and 40% in arm A and B, respectively, several findings are generated (Supplementary Simulation Study 1): (a) a one-to-one relationship between one-sided Fisher exact test (odds ratio [OR] < 1) and the unfavorable arm B’s prior (*c* = 1) (Supplementary Figures S1 and S2). This relationship has also been previously reported by Altham (1969) and Agresti and Hitchcock (2005) [18, 19]. (b) The three non-informative priors show a higher *Pr*(*B* > *A*) than the unfavorable arm B’s prior (*c* = 1) (Supplementary Figures S3 and S4). In terms of p value language, the three non-informative priors yield a smaller p value than the one-sided Fisher exact test. Also, the three non-informative priors have a similar *Pr*(*B* > *A*) (largest difference < 0.02; Supplementary Figures S5 and S6). (c) The *Pr*(*B* > *A*) decreases in the unfavorable arm B’s prior (Supplementary Figure S7), but increases in the favorable arm B’s prior (Supplementary Figure S8) as c increases from 0.1, 1, to 10. (d) Like the favorable arm B’s priors, the prior using the hypothesized or observed response rate yields a higher *Pr*(*B* > *A*) than the unfavorable arm B’s prior (*c* = 1) in most cases (Supplementary Figures S9 and S10).

**Figure 2:**
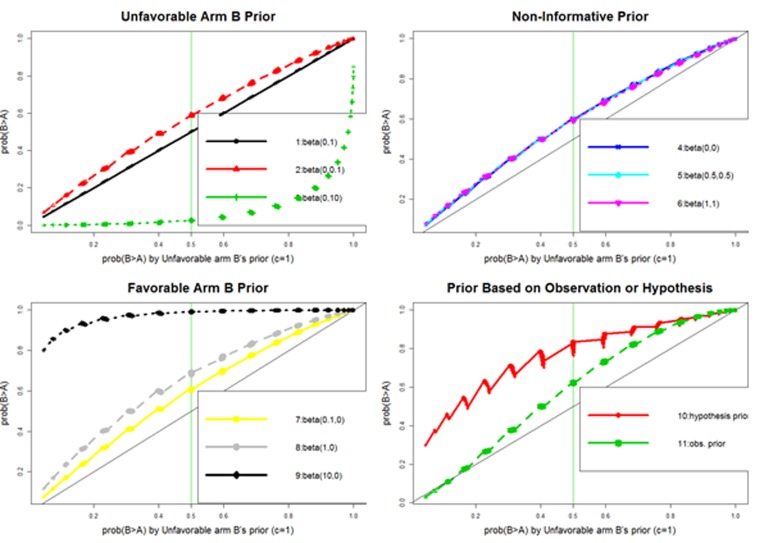
Comparison of various prior distributions regarding their impact on Bayesian posterior probability

In summary, prior distribution affects *Pr*(*B* > *A*) (Figure 2). Strong unfavorable or favorable treatment arm priors will give a large increase or decrease in *Pr*(*B* > *A*) while the non-informative priors give a small increase (i.e., a slight advantage favoring arm B). Thus, if preliminary data are limited or not available, the non-informative priors are preferable. Because the non-informative priors have a very similar *Pr*(*B* > *A*), the non-informative prior, *beta*(1,1), is used in both arms to calculate *Pr*(*B* > *A*) for determining the winner.

### Relation of *Pr*(*B* > *A*) and response rate difference

With the simulation setting A and the non-informative prior, *beta*(1,1), in both arms, results show an increasing pattern of *Pr*(*B* > *A*) as the difference of response rate increases (Supplementary Figure S11, A and B, and Supplementary Simulation Study 2). However, it is not a complete one-to-one relation. There is a small range of *Pr*(*B* > *A*) in each distinct response rate difference. One explanation is that there may be multiple ways to yield a same difference of response rate, thus changing the posterior beta distribution, *beta*(1+k,1+n-k), in each arm. As a result, *Pr*(*B* > *A*) is altered accordingly. Nevertheless, the difference is quite small ( < 0.02; Supplementary Figure S11B). Therefore, *Pr*(*B* > *A*) could be approximated to the response rate difference. In the simulation example, a Bayesian posterior probability of 80% is about 10% response rate difference.

### Delta effect (δ)

Delta (δ) is activated only when both arms are competitive. Thus, it affects power and type I error locally (i.e., in the case of both arms passing the second stage). Sensitivity analysis is performed under various conditions using the simulation setting A and the non-informative prior, *beta*(1,1), in both arms to select an acceptable threshold of δ to balance local power and type I error (Supplementary Simulation Study 3). For the impact on the local type I error, the proportion of both arms being competitive is 0.93% using the null hypothesis (20% response rate in both arms). Half of the 0.93% probability (0.47%) misclassify arm B as winner at δ = 0.5 (i.e., local type I error = 0.47%; Supplementary Table S1). As δ increases to 0.8 and 0.9, 0.01% or less are claiming arm B as winner (i.e., local type I error ≤0.01%). For the effect on local power, the probability of both arms being competitive is 9% under the alternative hypothesis (40% in arm B and 20% in arm A, a 20% difference of response rate). The local power to claim arm B as winner is 8%, 4%, and 2%, for δ = 0.5, 0.8, and 0.9, respectively (a 2-fold reduction of power from δ = 0.5 to 0.8, and a 2-fold reduction of power from δ = 0.8 to 0.9; Supplementary Table S1). In the 15% difference of response rate, power reduction is 2- to 3-fold from δ = 0.5 to 0.8. Two additional analyses (45% vs. 30% in Supplementary Table S2 and 15% vs. 5% in Supplementary Table S3) show a similar pattern of (1) 50% misclassification rate at δ = 0.5 and (2) 3- to 5-fold in power reduction from δ = 0.5 to 0.8 for 10% to 15% difference of response rate. In short, when both arms are competitive, a δ of 0.5 will cause a randomly selected winner (misclassification rate = 50%). For δ increasing to 0.8 or higher, the local type I error (at least 10 folds) will be decreased significantly, but the local power will be reduced moderately (2- to 5-fold) to detect a 10% to 20% difference of response rate. Although there is no a clear cutoff for δ, a δ of 0.8 is a reasonable choice to determine the winner.

### Our data example

In advanced non-small cell lung cancer (NSCLC), immunotherapy (e.g., anti-PD-L1 or anti-PD-1 agents) has shown a promising response rate of about 20% in recent phase I clinical trials [20-24]. However, about 80% of patients do not respond. To improve the efficacy, we designed a phase II trial at our institute to evaluate the efficacy of the combination of anti-PD-1 plus a histone deacetylase inhibitor (HDACi) [25-30] and compared single agent, anti-PD-1, in this targeted population. The trial has been registered in ClinicalTrials.gov (ID: NCT02638090). This study presents several statistical challenges: (1) the anti-PD-1 and the combination are experimental regimens in this population, (2) early termination of ineffective drug combination(s) is preferred to minimize unnecessary exposure to toxicity, and (3) randomization is favored to reduce selection bias.

### Trial design

With these considerations, we employed this Bayesian pick-the-winner design in a randomized phase II clinical trial. Both treatment arms were labeled as arm A for anti-PD-1 only and arm B for combination of anti-PD-1 and HDACi. From historical data in an unselected NSCLC population, we considered a 20% response rate or below as ineffective. We used 40% response rate as a promising result to further pursue future study. For each arm, using a Simon optimal two-stage design with initial 10% type I and II error rate, 17 patients will be enrolled in the first stage of the trial. If 3 or fewer patients respond, that treatment will be stopped. If 4 or more patients show a response, 20 additional patients (a total of 37 patients per group) will be enrolled in that arm. If the total number responding is 10 or less, we will conclude that the treatment is not effective.

If both arms fail at the first or second stage, the trial will be stopped. No winner will be claimed. The sample size will be 34 if both arms fail at the first stage and 54 if only one arm fails at the first stage. If only one arm passes the second stage, the arm will be the winner. If both arms pass the second stage, we will use *Pr*(*B* > *A*) to select the winner. The non-informative prior of beta distribution, *beta*(1,1), is used to calculate the posterior probability. Arm B will be claimed as the winner if *Pr*(*B* > *A*) > δ = 80% (about a 10% difference of response rate).

### Operating characteristics of the trial design by simulation

We were interested in the probability of *correctly* selecting an arm as superior to the other arm if it is truly superior (power) and, conversely, the probability of *incorrectly* selecting an arm that is no better than the other arm (type I error).

### Power

Assuming that the true probabilities of response in arms B and A are 40% and 20%, respectively (scenario 1: 20% difference of response rate), the overall probability (power) of correctly choosing arm B as superior is 86% on the basis of superiority shown at the end of the trial (Table 2). The probability of stopping arm A early and declaring arm B superior at the end of the trial is 82%. There is a 9% probability of both arms passing the second stage with 4% claiming arm B as the winner by the Bayesian posterior probability. In a 15% difference of response rate, the overall power is 71% and 75% for the comparison of arms B and A with 35% *versus* 20% (scenario 2) and 40% *versus* 25% (scenario 3), respectively (Table 2). The proportion of both arms passing the second stage is 7% in scenario 2 (scenario 3: 26%), with 2% (scenario 3: 11%) claiming arm B as the winner by the Bayesian posterior probability.

#### Type I error

In the null hypothesis of a 20% response rate in both arms, the overall probability (type I error) of incorrectly choosing arm B as superior is 9% (scenario 4, Table 2). There is only a 1% probability of both arms passing the second stage and less than 0.01% probability misclassifying arm B as the winner.

#### Summary

With δ = 0.8, the design has an 86% power to detect a 20% difference of response rate. The power slightly decreases but remains above 70% (71%-75%) to differentiate a 15% difference of response rate. The type I error is controlled at 9% when both arms have a 20% response rate.

### Comparison of bayesian posterior probability to fisher exact test

For this comparison, local power and type I error are evaluated between the Bayesian posterior probability and Fisher exact test in terms of winner determination when both arms pass the second stage. The decision rule is (i) δ = 0.8 with the non-informative prior, *beta*(1,1), in both arms for the Bayesian posterior probability, and (ii) a one-sided *p* < 0.05 for the Fisher exact test. Simulation results in Table 3 show that the added local power in claiming arm B as the winner is higher by the Bayesian posterior probability (2%-11%) than by the Fisher exact test (0.2%-2%). However, the local type I error is also higher in the Bayesian posterior probability (0.01%) compared with Fisher exact test ( < 0.01%).

### Comparison to a standard controlled randomized trial (CRT)

We compared the standard two-arm CRT without interim analysis with our approach in terms of overall power and type I error. With the same hypothesis testing of 40% *versus* 20% response rate and a sample size of 37 patients per arm, the CRT design gives a power of 62% for a one-sided type I error of 9% using the Fisher exact test. In comparison, the proposed Bayesian design achieves a higher power, 86%, detecting a 20% difference of response rate with a type I error of 9%.

### Applications in gonzalez-martin et al. and dark et al. studies [5, 6]

#### Pick-the-winner

Both studies have completed their two treatment arms. However, the winners were determined by the highest response rate and without formal statistical comparison. In the Gonzalez-Martinet al. study [6], the arm with combination of paclitaxel and carboplatin, labeled as arm B, was claimed as winner because of a higher response rate (31/38 = 81.5%) compared with the arm with carboplatin only, labeled as arm A (20/40 = 50%). With our approach and δ = 0.8, the posterior beta distribution will be *beta(1+31,1+7*) for arm B and *beta(1+20,1+20)* for arm A based on the non-informative prior, *beta(1,1)*. Accordingly, there is a 99.8% chance of arm B having a higher response rate than arm A (i.e., *Pr*(*B* > *A*) = 99.8% > δ); therefore, arm B is the winner. In the study of Dark et al., the arm with 1.8 mg OSI-211 was the winner because of a higher response rate (6/39 = 15%) compared with the arm with 2.4 mg OSI-211 (2/41 = 5%). With our approach, the corresponding Bayesian posterior probability of higher response rate in the arm with 1.8 mg OSI-211 than in the arm with 2.4 mg OSI-211 was 93% (i.e., *Pr*(*B* > *A*) = 93% > δ). Thus, our method shows the arm with 1.8 mg OSI-211 as the winner. In comparison, the Fisher exact test gives a p value of 0.003 and 0.12 (one-sided test) for Gonzalez-Martinet al. and Dark et al. studies, respectively.

### Power

Gonzalez-Martinet al. [6] compared a 45% *versus* 30% response rate (15% difference), whereas Dark et al. [5] compared response rates of 15% *versus* 5% (10% difference). Samples sizes for both studies were 38-41 patients per group. With the same hypotheses, the Bayesian pick-the-winner design requires 38 patients per arm and provides a 72% power to detect a 15% difference of response rate with a type I error of 12% for the Gonzalez-Martinet al. study (Supplementary Simulation Study 4). For the study of Dark et al., our approach needs 36 patients per arm and gives a 75% power to detect the 10% difference with a type I error of 9% (Supplementary Simulation Study 5).

## DISCUSSION

In this study, we developed an integrative design by incorporating Bayesian posterior probability with Simon two-stage design in a randomized phase II clinical trial. This approach makes the best use of the Bayesian method, Simon two-stage design, and randomization to identify an effective drug as the winner and to warrant a phase III trial for final evaluation. As a result, it has applied to two randomized phase II clinical trials with immunotherapy (NCT03071406 for Merkel cell skin cancer and NCT02638090 for lung cancer registered in ClinicalTrials.gov). Historically, a Simon two-stage design is widely used in a single arm setting. Here, we have taken advantage of its uniqueness of small sample size and early termination of ineffective drug(s) in a randomized trial design. The randomization strategy serves to balance unknown confounding factors, therefore reducing selection bias and creating a higher degree of comparability for objective comparison. Most importantly, utilization of Bayesian posterior probability provides a rigorously statistical mean to select the winner, in contrast to an arbitrary decision based on the highest response rate.

For clinical investigators, the Bayesian pick-the-winner design holds unique advantages when both arms successfully pass the second stage. It guarantees that a winner will be decided not just based solely on the highest response rate but also based on a sound formal statistical method. In an era of randomized phase III oncology clinical trials where numerous agents and combination therapies have failed to accomplish both statistically and clinically significant outcomes, having an additional safeguard to correctly move arms forward to larger trials is highly needed. This holds significance not only for clinicians but also for patients. By using a small sample size with early termination control of ineffective drugs, the Bayesian pick-the-winner method reduces the burden of trial costs and undue exposure to toxicity for our cancer patients. As key stakeholder in patient treatment algorithms, we have an obligation to continue to enrich and evolve the field of clinical research in a right direction.

Two types of power comparisons were performed in our data example. One used local power for the comparison between Bayesian posterior probability and one-sided Fisher exact test only when both arms passed the second stage. Simulation results showed that the Bayesian posterior probability contributed a larger local power, but also induced a higher local type I error. The other one used overall power for comparison to a standard CRT without interim analyses. Because a CRT is a one-stage design, direct comparison to the Bayesian posterior probability is not feasible. Therefore, overall power is used for comparison. Results indicated that the Bayesian pick-the-winner design gives a higher power to determine the winner given the same sample size and the type I error.

We showed two important points in regard to application of our design to the Gonzalez-Martinet al. and Dark et al. studies [5, 6]. One was the ability of the Bayesian pick-the-winner design to provide a reasonable power even with a small sample size of around 40 subjects per arm for a randomized trial. The other one was the utility of the Bayesian posterior probability to pick the winner among the competitive treatment arms.

Although the Bayesian posterior probability is a useful tool to pick the winner, two parameters are required: prior distribution and δ effect. Both factors need to be considered simultaneously in order to properly calculate power and type I error. For the prior distribution, the use of unfavorable treatment arm prior with *c* = 1 is equivalent to the classic one-sided Fisher exact test. Our evaluation in beta prior distribution indicates strong favorable or unfavorable treatment arm priors would have a high likelihood to accept or reject the treatment arm as the winner. The non-informative priors give a slight advantage in treatment arm and are preferred for use to avoid bias if preliminary data are limited. In addition, the Bayesian posterior probability could be approximated into the response rate difference and make its clinical interpretation more meaningful. For the δ effect, it affects local power and type I error. Sensitivity analysis showed that a δ of 0.5 will lead to a random choice of winner selection. When δ increases to 0.8 or higher, the local type I error will be substantially reduced, but a decrease in the local power is moderately controlled. The δ function is similar to the parameter d (a threshold parameter of response rate difference) in the approach of Sargent and Goldberg [31] (Supplementary Comparison 1).

The current Bayesian pick-the-winner design focuses on a two-arm setting using a Simon two-stage method. A R package, ‘BayesianPickWinner’, with graphical user interface is provided for clinicians to easily generate a statistical plan for their clinical trial protocols (Supplementary R package and https://github.com/dungtsa/BayesianPickWinner). The design has flexibility to (i) allow each arm using different parameters in Simon two-stage design or (ii) use different designs (e.g., Fleming single stage design [32] in Supplementary Simulation Study 6). However, we suggest the same parameters within the same design to simplify the trial procedure and make the implementation easier. The Bayesian pick-the-winner design could be extended to 3 or more treatments. If multiple arms pass the second stage, the Bayesian posterior probability can be easily calculated by comparing their posterior beta distributions using Monte Carlo simulation.

In summary, our integrative design pulls together Bayesian posterior probability, Simon two-stage design, and randomization into a unique setting. This design allows objective comparisons between treatment arms to determine the winner.

## MATERIALS AND METHODS

A pick-the-winner design developed for a two-arm randomized phase II clinical trial integrates three key components: Bayesian posterior probability, Simon two-stage design, and randomization. The goal is to objectively identify an effective treatment to warrant a future large phase III trial.

### A two-arm randomized simon two-stage design

Assume there are two treatment arms, A and B. They may be separate treatments placed to compete against each other, or treatment B is the combination of treatment A plus an additional drug. To balance unknown confounding factors, randomization is implemented to assign patients to the two treatment arms. For each treatment arm, a Simon two-stage design [1] is used to evaluate the efficacy of the treatment. In this design, when an arm passes the second stage, we consider this as a “*competitive*” arm. The winner could be easily determined if only one arm is competitive. However, when both arms are competitive, it becomes a challenging issue to objectively determine the winner. Although some arbitrary criteria could be used, such as difference of response rate or cost, these do not have statistical justification and could risk a false positive finding.

### Bayesian posterior probability

To address the issue of statistical comparisons when both arms are competitive, we integrated a Bayesian posterior probability into the two-arm randomized Simon two-stage design. The Bayesian posterior probability, *Pr*(*B* > *A*), is defined as probability of a response rate in arm B higher than in arm A. For arm A with a total sample size of *n*_A_ at the end of the second stage, the number of responses, *k*_A_, does not follow a binominal distribution, but its likelihood function has a form of θ *k*_A_(1- θ) *n*_A_-*k*_A_ where θ represents the response rate (Supplementary Method 1). Thus, given a beta prior distribution assumption with two parameters, a and b, (i.e., *beta*(*a,b*)), the posterior distribution of response rate becomes another beta distribution, *beta*(*a*+*k*_A_,b+*n*_A_-*k*_A_). Similarly, the posterior distribution for arm B will be *beta*(*a*+*k*_B_,b+*n*_B_-*k*_B_). *Pr*(*B* > *A*) therefore becomes a probability of *beta*(*a*+*k*_B_,b+*n*_B_-*k*_B_) > *beta*(*a*+*k*_A_,b+*n*_A_-*k*_A_), meaning the chance of response rate higher in arm B than in arm A (A R function code in Supplementary Method 2). This posterior probability is utilized when both arms are competitive. Arm B will be claimed as the winner if *Pr*(*B* > *A*) > δ and arm A will be claimed as the winner if *Pr*(*B* > *A*) < (1-δ). Because specification of prior distribution will affect *Pr*(*B* > *A*) and δ will influence the corresponding power and type I error, both issues are evaluated in the simulation study.

### Algorithm of the bayesian pick-the-winner design

For each arm, a Simon two-stage design (optimal or min-max) with an initial type I error of α and a type II error of β is used to calculate sample size as follows: There will be n1 patients enrolled in the first stage of the trial. If *r*_1_ or fewer patients respond, the treatment will be stopped. If *r*_1_+*1* or more patients show a response, *n*_2_ additional patients (a total of *n*_1_+ *n*_2_ patients per arm) will be enrolled. If the total number of responders is r or less, we can conclude that the treatment is ineffective. If both arms fail at the first or second stage, the trial is stopped (Table 1). No winner will be claimed. If there is only one competitive arm, the competitive arm will be the winner. If both arms are competitive, we can use *Pr*(*B* > *A*) to select the winner. Arm B will be claimed as the winner if *Pr*(*B* > *A*) > δ. The power and type I error for the treatment comparison are determined by simulation in alternative and null hypotheses. A flow chart of the algorithm is shown in Figure 1.

**Table 1:** Rules to pick the winner

		Arm B		
		fail in stage 1	fail in stage 2	pass stage 2
**Arm A**	**fail in stage 1**	both losers	both losers	Arm B winner
	**fail in stage 2**	both losers	both losers	Arm B winner
	**pass stage 2**	Arm A winner	Arm A winner	Arm B winner if *Pr(B>A)*> δ Arm A winner if *Pr(B>A)*< (1- δ)

Pr(B>A): posterior probability of the response rate in arm B higher than in arm A

**Table 2:** Operating characteristics

Scenario 1: Arm B vs. A: 40% *versus* 20%		Arm B		
	Probability	fail in stage 1	fail in stage 2	pass stage 2
**Arm A**	**fail in stage 1**	0.03	0.03	0.5
	**fail in stage 2**	0.02	0.02	0.32
	**pass stage 2**	0	0	0.09
Both arms passing the 2nd stage: 9%. Among them, Arm B claims 4.09% as winner. Overall power of Arm B= 86%.				

Scenario 2: Arm B vs. A: 35% *versus* 20%		Arm B		
	Probability	fail in stage 1	fail in stage 2	pass stage 2
**Arm A**	**fail in stage 1**	0.06	0.07	0.42
	**fail in stage 2**	0.04	0.05	0.27
	**pass stage 2**	0.01	0.01	0.07
Both arms passing the 2nd stage: 7%. Among them, Arm B claims 2.12% as winner. Overall power of Arm B= 71%.				

Scenario 3: Arm B vs. A: 40% *versus* 25%		Arm B		
	Probability	fail in stage 1	fail in stage 2	pass stage 2
**Arm A**	**fail in stage 1**	0.02	0.02	0.32
	**fail in stage 2**	0.02	0.02	0.33
	**pass stage 2**	0.01	0.01	0.26
Both arms passing the 2nd stage: 26%. Among them, Arm B claims 10.79% as winner. Overall power of Arm B= 75%.				

Scenario 4: Arm B vs. A: 20% *versus* 20%		Arm B		
	Probability	fail in stage 1	fail in stage 2	pass stage 2
**Arm A**	**fail in stage 1**	0.3	0.2	0.05
	**fail in stage 2**	0.19	0.13	0.03
	**pass stage 2**	0.05	0.03	0.01
Both arms passing the 2nd stage: 1%. Among them, Arm B claims 0.01% as winner. Type I error= 8.73%.				

**Table 3:** Comparison of Bayesian posterior probability and Fisher exact test regarding local power and type I error

			Probability of arm B as winner	
	Difference	Arm B vs. A	Bayesian posterior probability	Fisher exact test (one-sided)
**Power**	20%	40% vs. 20%	4%	0.7%
	15%	40% vs. 25%	11%	2%
		35% vs. 20%	2%	0.2%
**Type I error**	0%	20% vs. 20%	0.01%	<0.01%

## SUPPLEMENTARY MATERIALS FIGURES AND TABLES


