# Supplementary material for: A Bayesian pick-the-winner design in a randomized phase II clinical trial
Source: Oncotarget. 2017 Jul 7;8(51):88376–85. doi: 10.18632/oncotarget.19088 (PMC5687612; doi:10.18632/oncotarget.19088)
Supplement: Supplementary file 1 [file oncotarget-08-88376-s001.pdf]

Supplementary Materials

A Bayesian Pick-The-Winner Design in A Randomized Phase II Clinical Trial

Chen et al

Contents

Method 1: Posterior Distribution of the Response Probability 3

Method 2: Calculation of Bayesian Posterior Probability, Pr(B>A), by Monte Carlo Simulation 4

Simulation Study 1: Effect of Prior Distribution 5

Simulation scheme: 5

Simulation results: 5

i. Relationship between one-sided Fisher exact test (odds ratio, OR,<1) and unfavorable arm B's prior (*c*=1): 6

ii. Comparison of three non-informative priors versus the unfavorable arm B's prior (*c*=1): 7

iii. Impact of *c* in unfavorable arm B's prior: 12

iv. Impact of *c* in favorable arm B's prior: 13

v. Impact of prior using the hypothesized or observed response rate: 14

Summary: 16

Simulation Study 2: Relationship of the Bayesian Posterior Probability And Response Rate Difference 18

Simulation Study 3: Delta Effect on Local Power and Type I Error 20

Simulation Study 4: Comparison of 45 % Versus 30 % Response Rate 22

Sample Size Calculation 22

Operating Characteristics 22

Power Analysis 22

Type I error 23

Summary 23
Tables of Power Anlaysis 23
Simulation Study 5: Comparison of 15 % Versus 5 % Response Rate 25
Sample Size Calculation 25
Operating Characteristics 25
Power Analysis 25
Type I error 26
Summary 26
Tables of Power Anlaysis 26
Simulation Study 6: Fleming Single Stage Design (A prototype) 28
Comparison 1: Comparison of the Delta, δ, **in Bayesian Pick-the-Winner Design and the Difference of Response Rates, d, in the Sargent And Goldberg's Method** 31
R package 'BayesianPickWinner**":** 33

Method 1: Posterior Distribution of the Response Probability

The number of responses at the end of a trial does not follow a binomial distribution if there is an interim analysis. However, with a beta prior, the posterior distribution of the response probability does have the beta distribution.

Proof:

Assume n_1 subjects are enrolled with the number of responses, X_1, in the 1st stage. We also assume X_1 follow a binomial distribution with a response rate, θ. Similarly, in the 2nd stage, there are n_2 subjects enrolled with the number of responses, X_2, which follows a binomial distribution with the same response rate, θ. The 2nd stage is activated only if $X_1>=S_1$. When Y ($=X_1+X_2$, total number of responses) is at least S ($S>S_1$), the treatment is considered promising. Thus, the probability function, $P(Y=k|\theta,X_1>=S_1,k>=S)$, for $k=S, S+1,\ldots,n_1+n_2$., has the following form.

$$\mathrm{P}(\mathrm{Y}=k|\theta\,,\mathrm{X}_1>=\mathrm{S}_1,\mathrm{k}>=\mathrm{S})=\frac{1}{C}\times\sum_{x=\max(s_1,k-n_2)}^{\min(k,n_1)}P(X_1=x,X_2=k-x|\theta)$$

$$=\frac{1}{C}\times\sum_{x=\max(s_1,k-n_2)}^{\min(k,n_1)}\binom{n_1}{x}\theta^x(1-\theta)^{n_1-x}\binom{n_2}{k-x}\theta^{k-x}(1-\theta)^{n_2-k+x}$$

$$=\frac{1}{C}\times\sum_{x=\max(s_1,k-n_2)}^{\min(k,n_1)}\binom{n_1}{x}\binom{n_2}{k-x}\theta^k(1-\theta)^{n_1+n_2-k}$$

$$=\theta^k(1-\theta)^{n_1+n_2-k}\times\frac{1}{C}\times\left(\sum_{x=\max(s_1,k-n_2)}^{\min(k,n_1)}\binom{n_1}{x}\binom{n_2}{k-x}\right)$$

where $C=\sum_{k=S}^{n_1+n_2}\mathrm{P}(\mathrm{Y}=k|\theta\,,\mathrm{X}_1>=\mathrm{S}_1,\mathrm{k}>=\mathrm{S})$.

While this probability function is no longer a binominal distribution, but it has a form of $\theta^k(1-\theta)^{(n1+n2-k)}$. Thus, with a beta prior, beta(a,b), the posterior distribution of θ remains a beta distribution, beta(a+k,n1+n2-k+b).

Method 2: Calculation of Bayesian Posterior Probability, Pr(B>A), by Monte Carlo Simulation

Approach:
Posterior distribution in both arms follows beta distribution. So we can simulate response rate data based on the posterior beta distribution in each arm, say over 10,000 times. Then we will obtain Pr(B>A) by calculating the proportion of response rate in Arm B greater than in Arm A. This can be easily implemented in R.

R code:

```
Bayesian_posterior_probability <-function(n_response_armA, n_nonresponse_armA,
n_response_armB, n_nonresponse_armB,sim.n=100000)
{
  # number of response in Arm A: n_response_armA
  # number of non-response in Arm A: n_nonresponse_armA
  # number of response in Arm B: n_response_armB
  # number of non-response in Arm B: n_nonresponse_armB
  # n.sim: the number of simulations

  response_rate_A <-rbeta(sim.n,  1 + n_response_armA,
                        1 + n_nonresponse_armA);

  response_rate_B <- rbeta(sim.n, 1 + n_response_armB,
                         1 + n_nonresponse_armB)

  mean(response_rate_B > response_rate_A)  # ---- this is Bayesian posterior
probability, Pr(B>A)

}
```

```
# example:
Bayesian_posterior_probability(n_response_armA=15, n_nonresponse_armA=22,
n_response_armB=24, n_nonresponse_armB=13, prior_a_armA=1, prior_b_armA=1,
prior_a_armB=1, prior_b_armB=1,sim.n=100000)
[1] 0.98062
```

Simulation Study 1: Effect of Prior Distribution

A series of prior distributions are evaluated: (i) non-informative prior: beta(c,c) in both arms for *c*=0 (Haldane prior), 0.5 (Jeffreys prior), and 1 (Bayes prior), (ii) unfavorable arm B's prior: beta(*c*,0) in arm A and beta(0,*c*) in arm B for *c*=0.1, 1, and 10, (iii) favorable arm B's prior: beta(0,*c*) in arm A and beta(*c*,0) in arm B for *c*=0.1, 1, and 10, (iv) prior beta distribution based on hypothesized or observed response rate with standard deviation (SD) of 0.1 (e.g., for a comparison of 40% in arm B versus 20% in arm A, the prior beta distribution based on the hypothesized response rate will have a mean of 40% and 20% for arm B and A, respectively, with SD=0.1).

Simulation scheme:
The following simulation settings are used to evaluate the effect of prior distribution.

- Simon optimal two-stage design with H0: 20% response rate, H1: 40% response rate, 10% type I and II error for each arm.
- Sample size (n): a total of 37 subjects per arm. Specifically, each arm has a sample size of 17 patients in the 1st stage. If 4 or more patients show a response, the arm will continue to the 2nd stage with 20 additional patients. An arm with a total number of responders greater than 10 is considered as 'competitive' (i.e., passing the 2nd stage).
- Hypothesized response rate in each arm: 20% in arm A and 40% in arm B.
- Simulation times: 100,000
- Bayesian posterior probability of response rate higher in arm B than in arm A (prob(B>A)) (see Method 2).
- Prior beta distribution based on the hypothesized or observed response rate with standard deviation (SD) of 0.1: the parameters (a and b) for the prior beta distribution is estimated by the function code, 'estBetaParams' (a conversation from mean and variance (SD^2) to the two parameters, a and b).

```
estBetaParams <- function(mu, var) {
  alpha <- ((1 - mu) / var - 1 / mu) * mu ^ 2
  beta <- alpha * (1 / mu - 1)
  ans<-c(alpha,beta)
  names(ans)<-c('alpha','beta')
  ans
}
```

Simulation results:

Simulation analysis is conducted in multiple ways: (i) relationship between one-sided Fisher exact test (odds ratio, OR,<1) and unfavorable arm B's prior (*c*=1), (ii) comparison of three non-informative priors versus the unfavorable arm B's prior (*c*=1), (iii) impact of *c* in unfavorable arm B's prior, (iv) impact of *c* in favorable arm B's prior, (v) impact of prior using the hypothesized or observed response rate.

i. Relationship between one-sided Fisher exact test (odds ratio, OR,<1) and unfavorable arm B's prior (*c*=1):

Comparison in Figure S1 shows the Bayesian posterior probability using the unfavorable arm B's prior (c=1) is equal to (1-p value of one-sided Fisher exact test (OR<1)). While both numerical probabilities are not exactly identical, the difference is quite small, likely due to simulation error (the largest difference=0.006; SD of the difference=0.001; Figure S2). Literatures also support this relationship by Altham (1969), and Agresti and Hitchcock (2005) ((Altham 1969, Agresti and Hitchcock 2005)). Because of this property, the unfavorable arm B's prior (c=1) is used as the baseline for the next comparisons.

Figure S1:

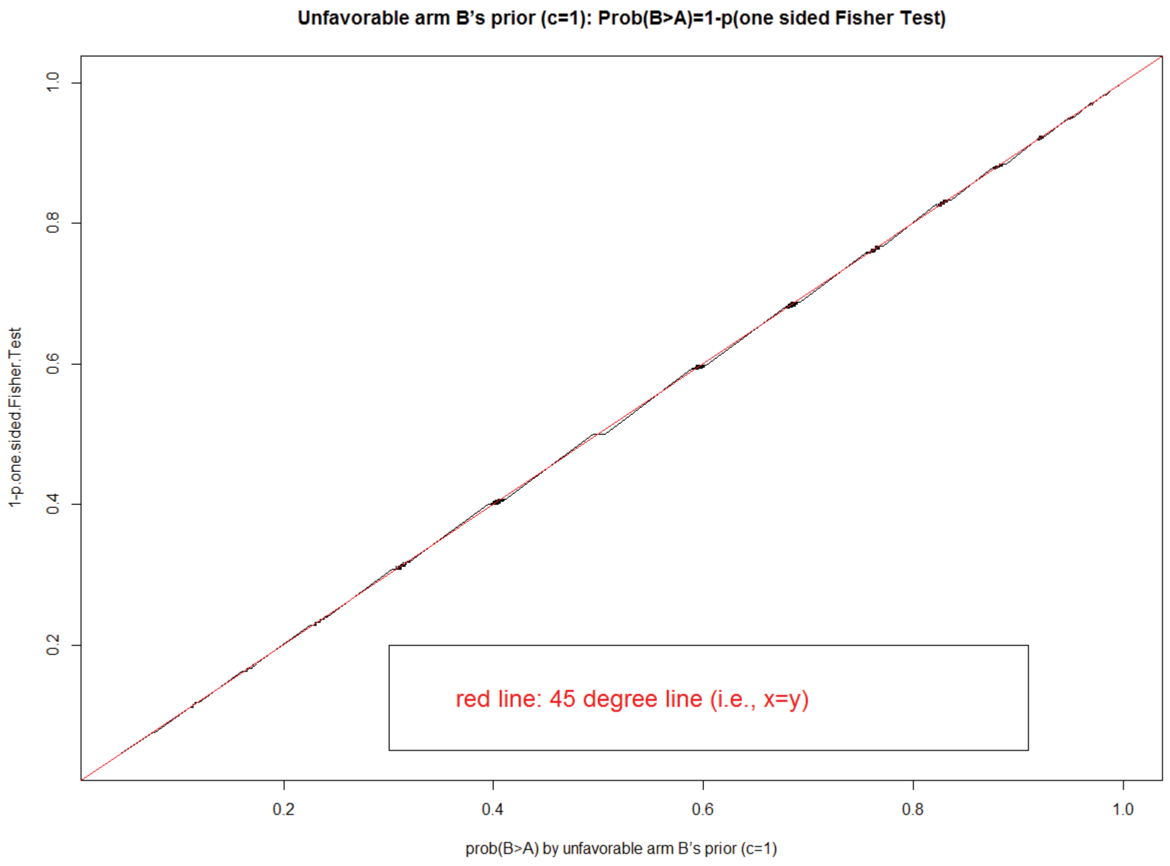

Figure S2

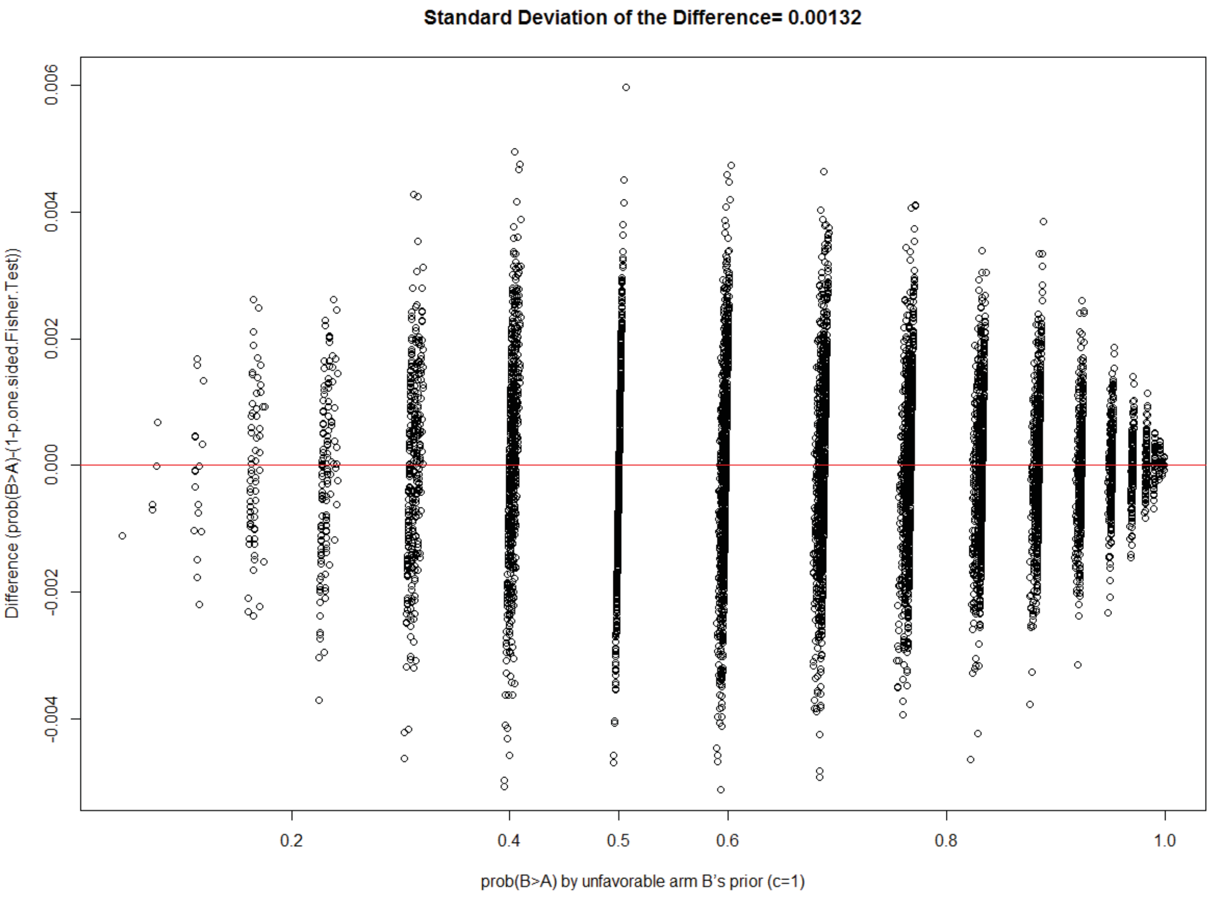

ii. Comparison of three non-informative priors versus the unfavorable arm B's prior (*c*=1):
We evaluate three non-informative priors, beta(c,c), with c=0, 0.5, and 1, for both arms and compare to the unfavorable arm B's prior (*c*=1). Results in Figure S3 show the Bayesian posterior probability is higher with the non-informative priors than with the unfavorable arm B's prior (*c*=1). In other words, the three non-informative priors give a higher Bayesian posterior probability compared to the one with prior for one-sided Fisher exact test. In term of p value language, the non-informative priors generate a smaller p value than the one-sided Fisher exact test.
The difference of the three non-informative priors versus the unfavorable arm B's prior (Figure S4) shows a 'n' shape. Specifically, when the unfavorable arm B's prior has the Bayesian posterior probability in the range of 30%-70%, the Bayesian posterior probability with the three non-informative priors gains 7%-11% more. In a range of 80%-90%, the gain for the non-informative priors is 3%-7%.

Difference of the Bayesian posterior probability among the three non-informative priors is minor with the largest difference of 0.0137 (SD=0.0024; 97.1%<0.01; Figure S5). Interestingly, with the beta(0,0) prior as the baseline, when its Bayesian posterior probability is less than 50%, the Bayesian posterior probability increases from c=0 to 1 (Figure S6). On the other hand, the Bayesian posterior probability decreases from c=0 to 1 when the Bayesian posterior probability using the beta(0,0) as prior is greater than 50%.

Figure S3:

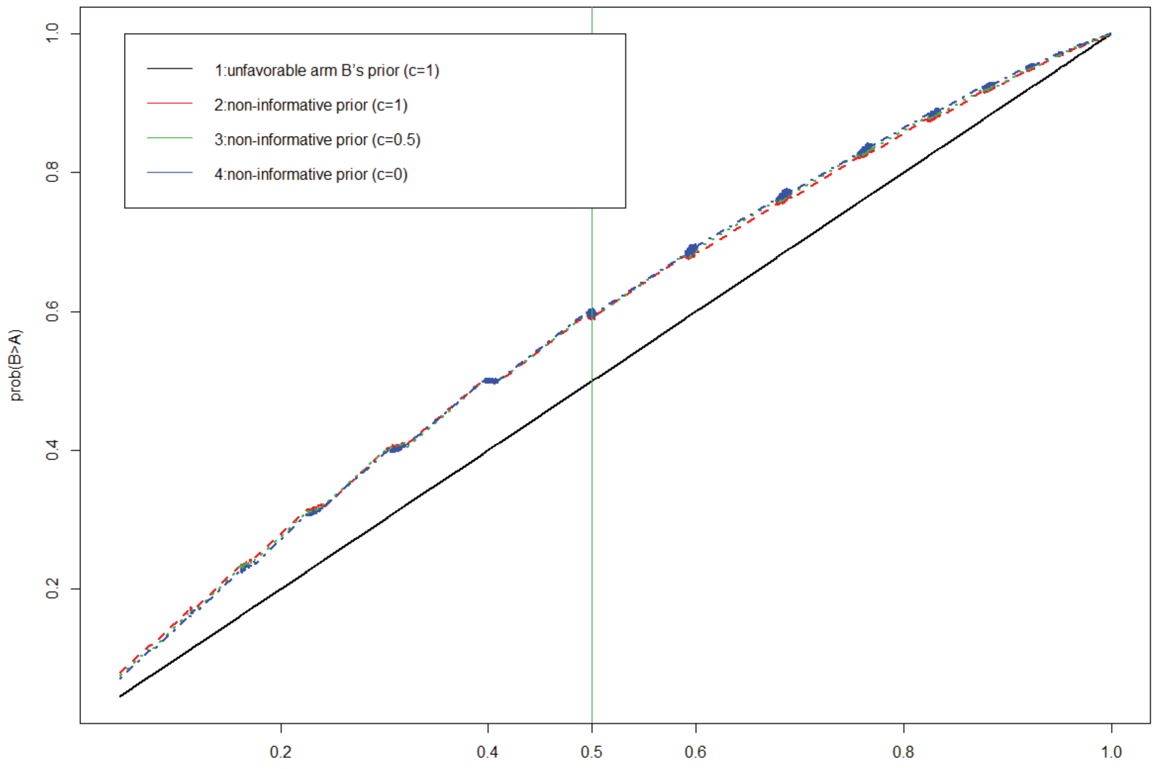

Figure S4:

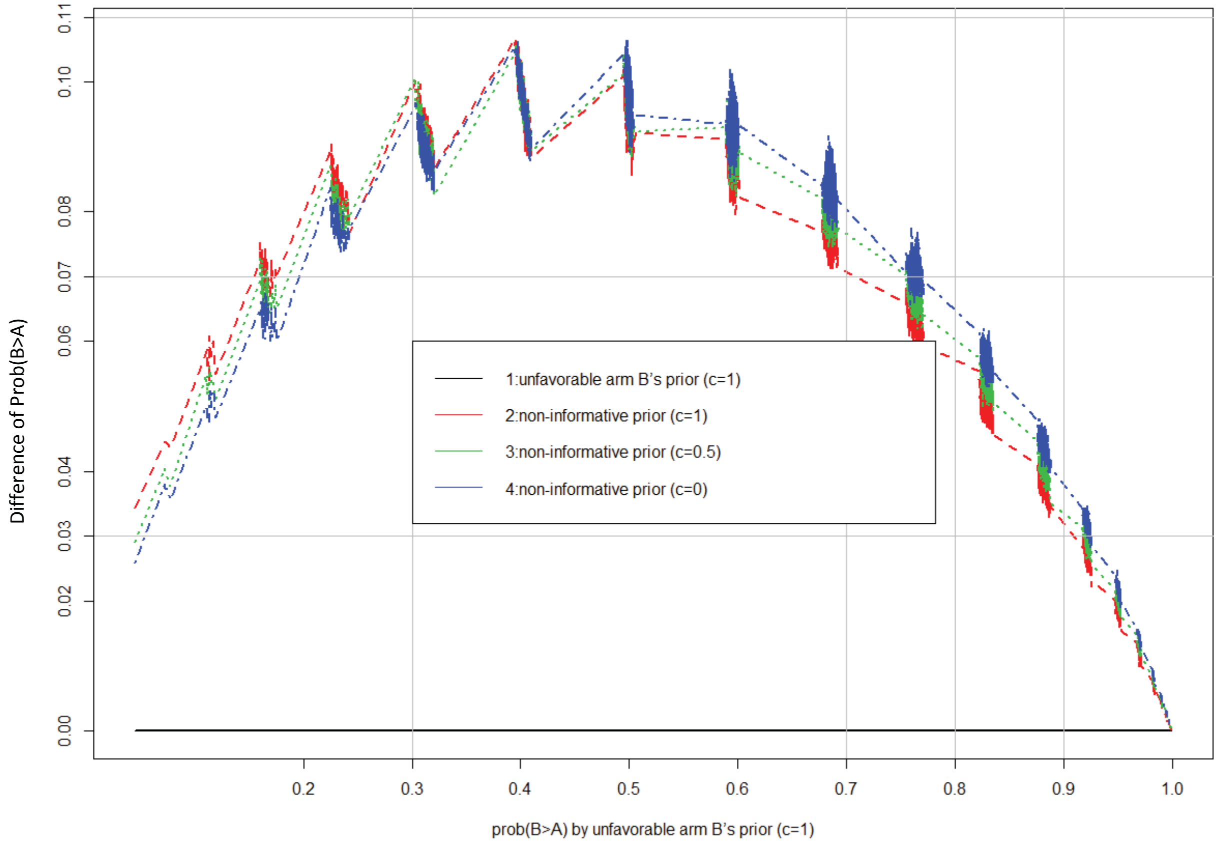

1:unfavorable arm B's prior (c=1)
2:non-informative prior (c=1)
3:non-informative prior (c=0.5)
4:non-informative prior (c=0)
Difference of Prob(B>A)
0.00
0.02
0.03
0.04
0.06
0.07
0.08
0.10
0.11
0.2
0.3
0.4
0.5
0.6
0.7
0.8
0.9
1.0
prob(B>A) by unfavorable arm B's prior (c=1)

Figure S5:

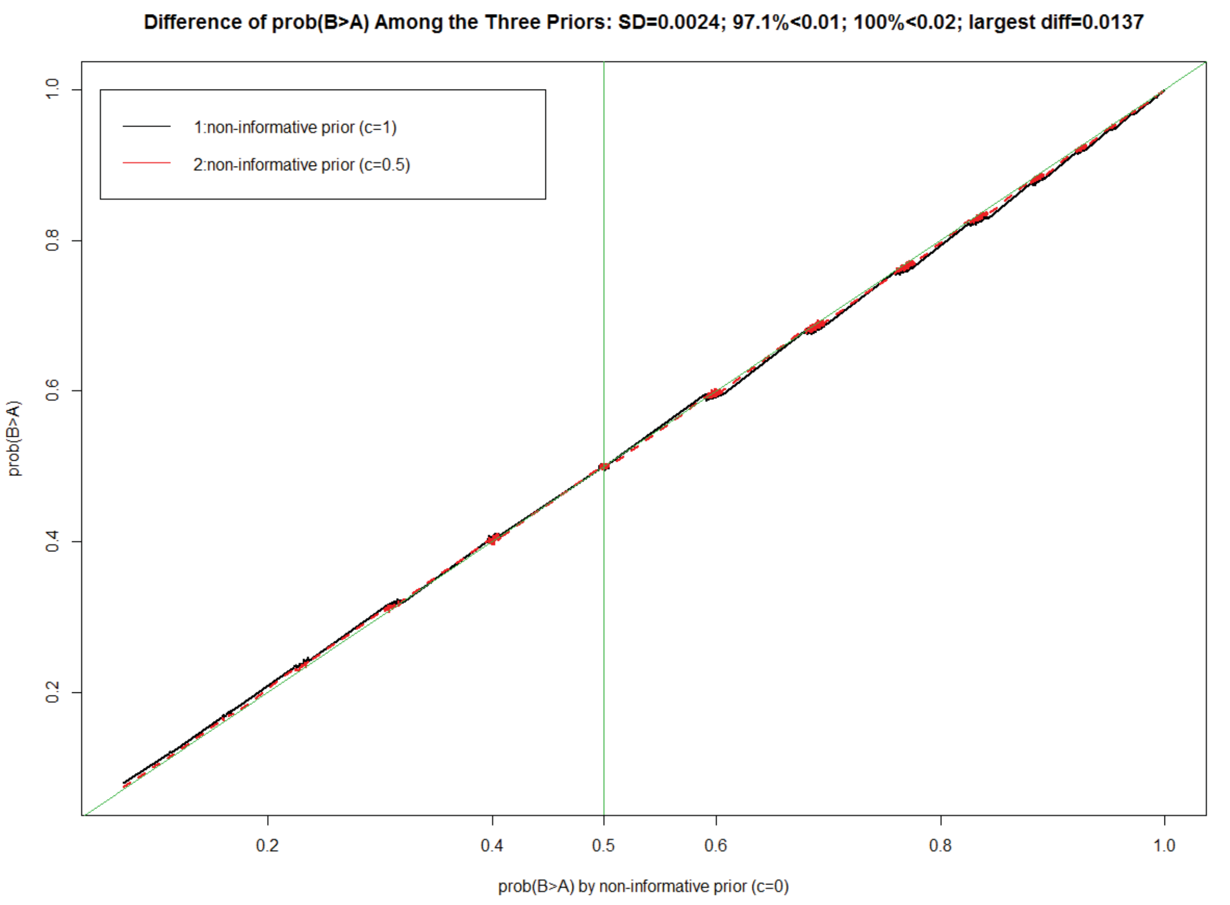

Difference of prob(B>A) Among the Three Priors: SD=0.0024; 97.1%<0.01; 100%<0.02; largest diff=0.0137
1:non-informative prior (c=1)
2:non-informative prior (c=0.5)
prob(B>A)
0.2
0.4
0.6
0.8
1.0
0.2
0.4
0.5
0.6
0.8
1.0
prob(B>A) by non-informative prior (c=0)

Figure S6:

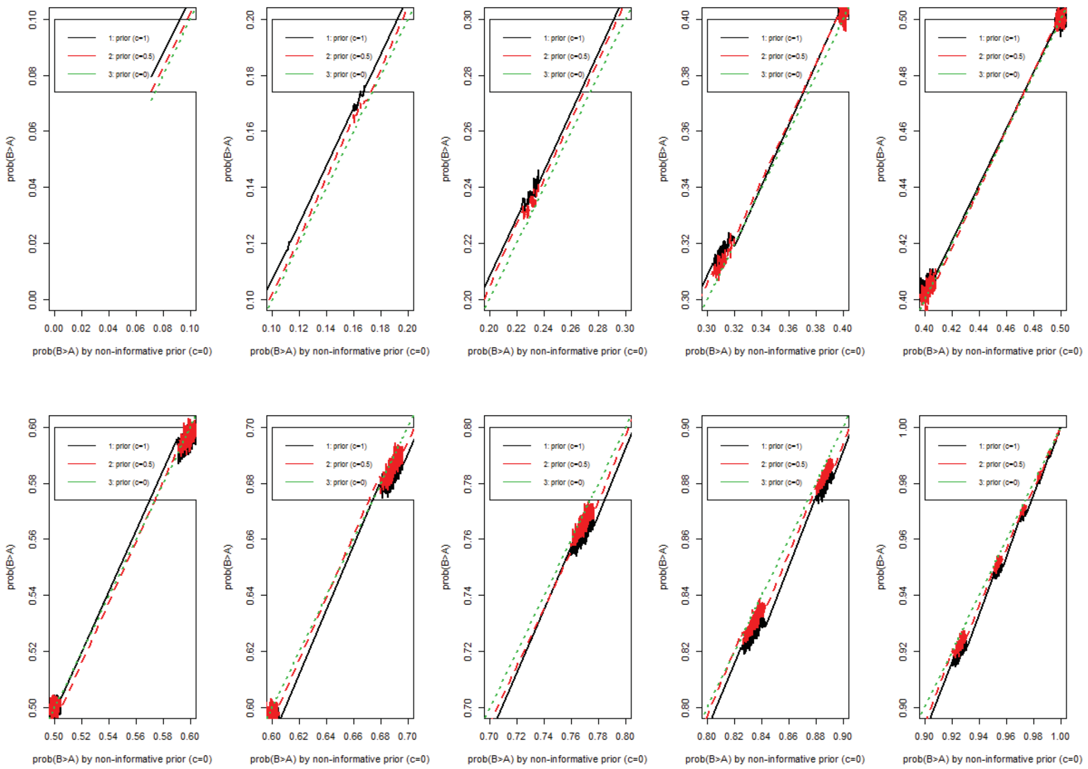
1: prior (c=1)
2: prior (c=0.5)
3: prior (c=0)
prob(B>A)
prob(B>A) by non-informative prior (c=0)

iii. Impact of c in unfavorable arm B's prior:

The Bayesian posterior probability decreases as c increases from 0.1, 1, to 10, for the unfavorable arm B's prior (Figure S7). For c =10, its Bayesian posterior probability is below 25% when the Bayesian posterior probability is 90% with c=1. When c is close to 0, such as 0.1, the Bayesian posterior probability is close to the non-informative prior with c=0.

Figure S7:

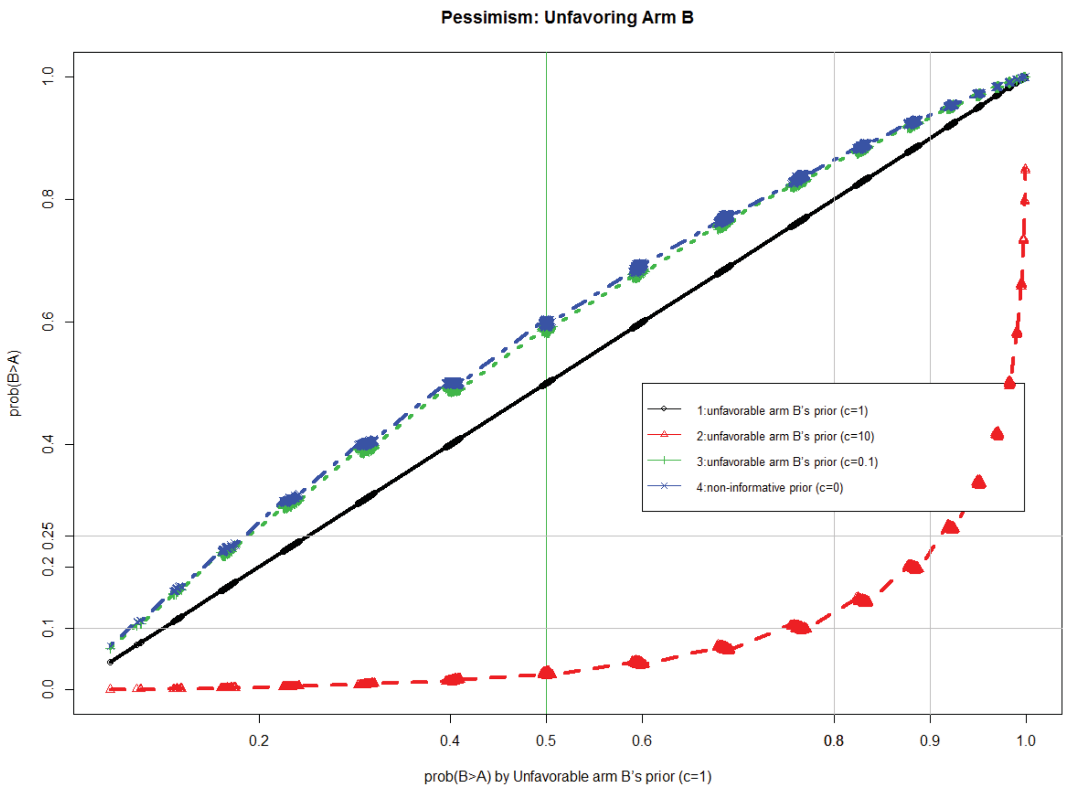

iv. Impact of *c* in favorable arm B's prior:

All favorable arm B's priors have a higher Bayesian posterior probability, as compared to the unfavorable arm B's prior *(c=1)* (Figure S8). The Bayesian posterior probability increases as *c* increases from 0.1, 1, to 10. For *c*=10, its Bayesian posterior probability reaches close to 90% while the Bayesian posterior probability is only 10% by the unfavorable arm B's prior. When *c* is close to 0, such as 0.1, the Bayesian posterior probability is close to the non-informative prior with *c*=0.

Figure S8:

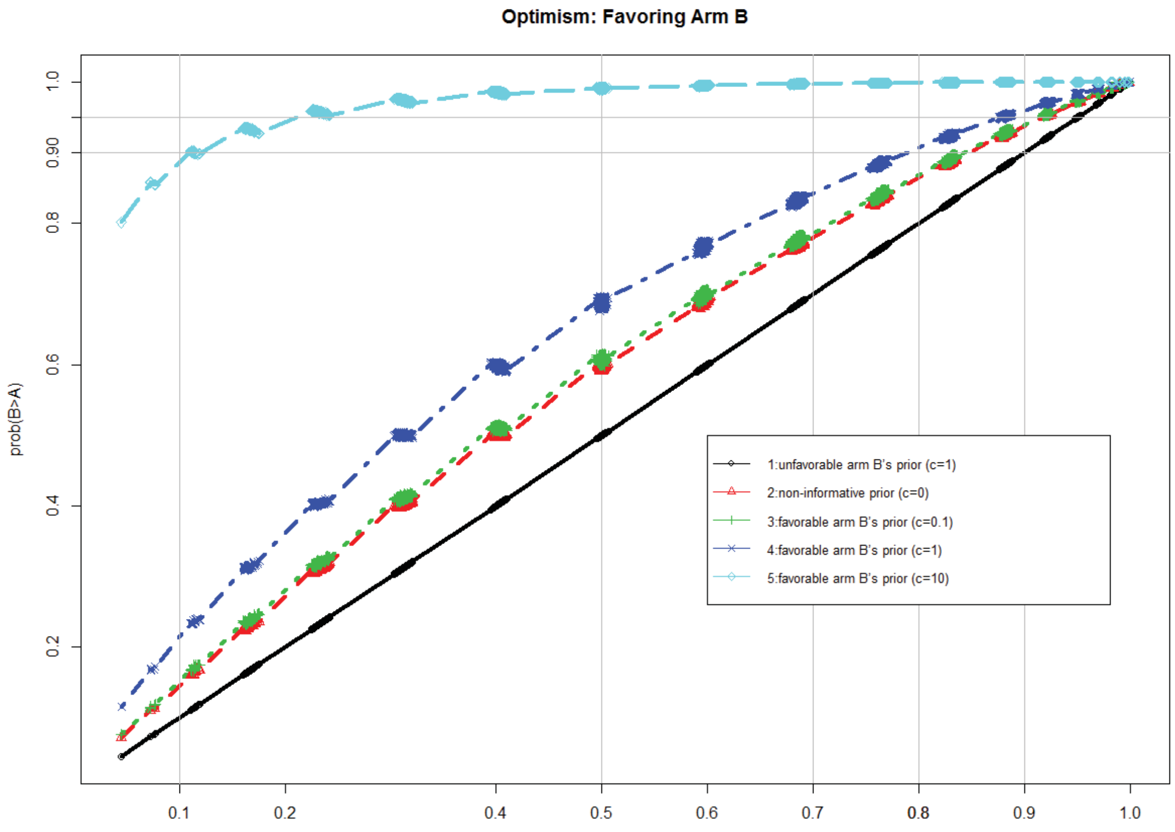

v. Impact of prior using the hypothesized or observed response rate:
With the hypothesized response rate of 20% in arm A and 40% in arm B as the prior mean and a prior SD of 0.1, the corresponding prior parameters (a and b) of beta distribution will be a=3 and b=12 for arm A and a=9.2 and b=13.8 for arm B using the function code 'estBetaParams'. When the Bayesian posterior probability by the unfavorable arm B's prior (*c=1*) is 80%, the probability with prior based on the hypothesized response rate reaches at least 90% (more than 10% difference; Figure S9-10). For a Bayesian posterior probability of 90% using the unfavorable arm B's prior, the prior based on the hypothesized response rate gains more than 5% power. In comparison to favorable arm B's priors, the Bayesian posterior probability is higher than the favorable arm B's prior (*c=1*), but less than the one with *c*=10 (Figure S11).

For the prior based on the observed response rate, when the response rate is substantially large in arm A compared to arm B (e.g., difference of response rate>10%), the Bayesian posterior probability will be smaller compared to the unfavorable arm B's prior (*c=1*). This could be seen in Figure S9-10 when the Bayesian posterior probability is 10% or less by the unfavorable arm B's prior (*c=1*). In other words, the prior based on the observed response rate will boost its favor in arm A if arm A outperforms arm B in terms of response rate. On the other hand, when the response rate is smaller or slightly large in arm A compared to arm B, the prior based on the observed response rate will have a higher Bayesian posterior probability than the unfavorable arm B's prior (*c=1*). This could be seen in Figure S9-10 when the Bayesian posterior probability is 20% or more by the unfavorable arm B's prior (*c=1*). In particular, if arm B outperforms arm A in terms of response rate, the prior based on the observed response rate will increase the probability to the same level or higher to the prior based on the hypothesized response rate, as shown in Figure S9-10 when the Bayesian posterior probability is 85% or larger by the unfavorable arm B's prior (*c=1*).

Figure S9:

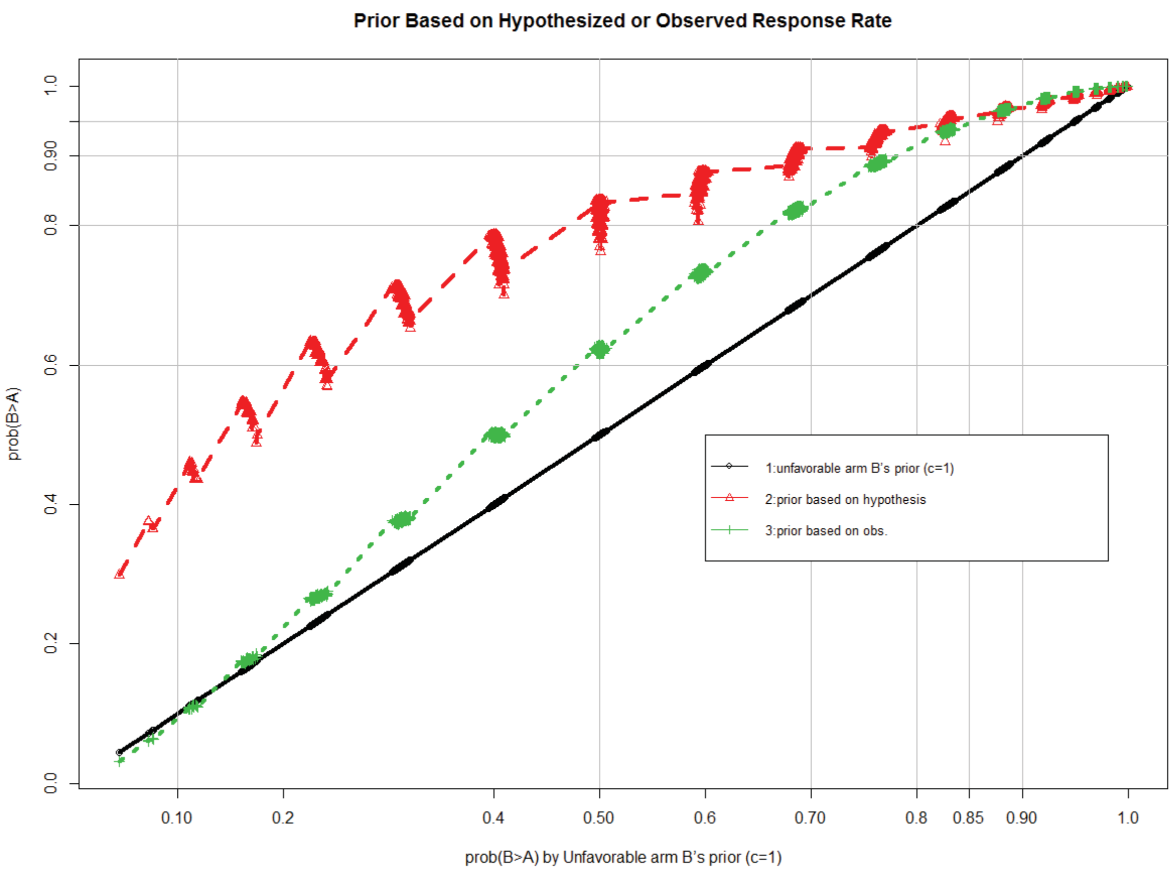

Prior Based on Hypothesized or Observed Response Rate
prob(B>A)
0.0
0.2
0.4
0.6
0.8
0.90
1.0
0.10
0.2
0.4
0.50
0.6
0.70
0.8
0.85
0.90
1.0
prob(B>A) by Unfavorable arm B's prior (c=1)
1:unfavorable arm B's prior (c=1)
2:prior based on hypothesis
3:prior based on obs.

Figure S10:

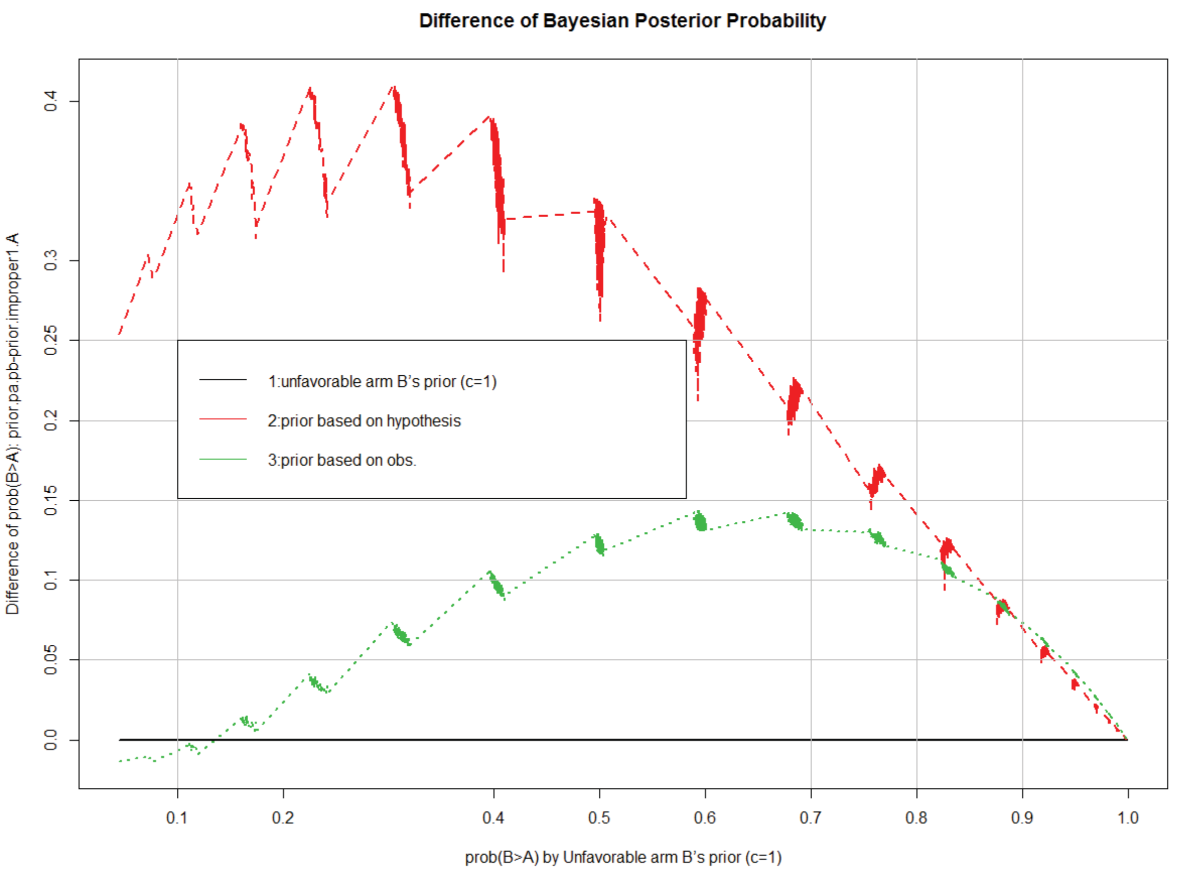

Summary:

- Bayesian posterior probability using the unfavorable arm B's prior (*c=1*) is equal to (1-p value of one-sided Fisher exact test (OR<1 where OR=odds in arm A/odds in arm B)).
- The three non-informative priors have a higher Bayesian posterior probability than the unfavorable arm B's prior (*c=1*).
- Difference of the Bayesian posterior probability in the three non-informative priors is less than 0.02.
- Bayesian posterior probability decreases as *c* increases from 0.1, 1, to 10, for the unfavorable arm B's prior.
- All favorable arm B's priors have a higher Bayesian posterior probability than the unfavorable arm B's prior (*c=1*). Also, the Bayesian posterior probability increases as *c* increases from 0.1, 1, to 10.
- Like the favorable arm B's priors, the prior using the hypothesized response rate has a higher Bayesian posterior probability than the unfavorable arm B's prior

(*c=1*). The prior based on the observed response rate also gives a higher Bayesian posterior probability to favor arm B if the response rate is smaller or not substantially larger in arm A than in arm B. Otherwise, it will favor arm A.

In summary, prior distribution definitely affects Bayesian posterior probability (Figure 2). With a pessimism prior (e.g., unfavorable arm B's prior), it will decrease the Bayesian posterior probability. If an optimism prior (e.g., the favorable arm B's priors, non-informative prior, or the prior using the hypothesized or observed response rate) is given, the probability will be increased. For the favorable arm B's priors and the prior using the hypothesized response rate, they will increase the Bayesian posterior probability relatively large. The prior using the observed response rate also gives a similar higher Bayesian posterior probability if the response rate is smaller or not substantially larger in arm A than in arm B. In contrast, the non-informative priors give a mild increase (i.e., a slight advantage in treatment arm). Thus, if preliminary data are limited or not available, the non-informative priors are preferred for use to avoid bias. Since difference of the Bayesian posterior probability among the three non-informative priors is minor (<0.02), the non-informative prior with beta(1,1) is used as the prior for calculating Bayesian posterior probability to determine the winner.

Figure 2:

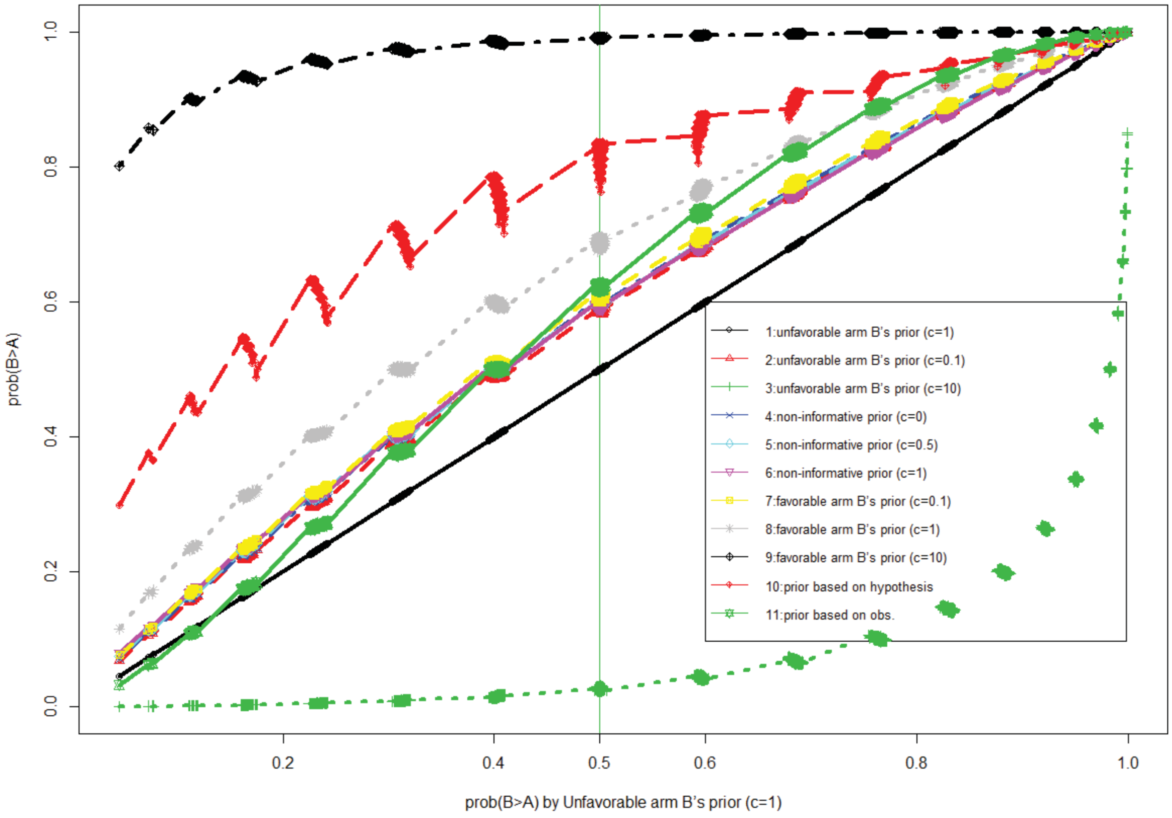

Simulation Study 2: Relationship of the Bayesian Posterior Probability And Response Rate Difference

Using the simulation results with beta(1,1) as the prior in Simulation Study 1, it shows an increasing pattern of Bayesian posterior probability as the difference of response rate increases (Fig S11A). However, it is not a complete one-to-one relationship. As shown in Fig S11A, there is a small range of Bayesian posterior probability in each distinct response rate difference. One explanation is that there may be multiple ways to yield the same difference of response rate, thus, changing the posterior beta distribution, *beta*(1+k,1+n-k), in each arm. As a result, the Bayesian posterior distribution is altered accordingly. For example, the design yields 8 ways for a difference of 4 responses (11% response rate difference) from 11 responses in arm A and 15 responses in arm B to 18 responses in arm A and 22 responses in arm B. The corresponding Bayesian posterior probability gives a minor variation with a range from 0.82 to 0.835 (Fig S11B). Nevertheless, the difference is quite small (<0.02). Therefore, it is reasonable to assume that the Bayesian posterior probability could be used to equate the response rate difference. In this simulation example, a Bayesian posterior probability of 80% is approximately 10% response rate difference.

Figure S11A:

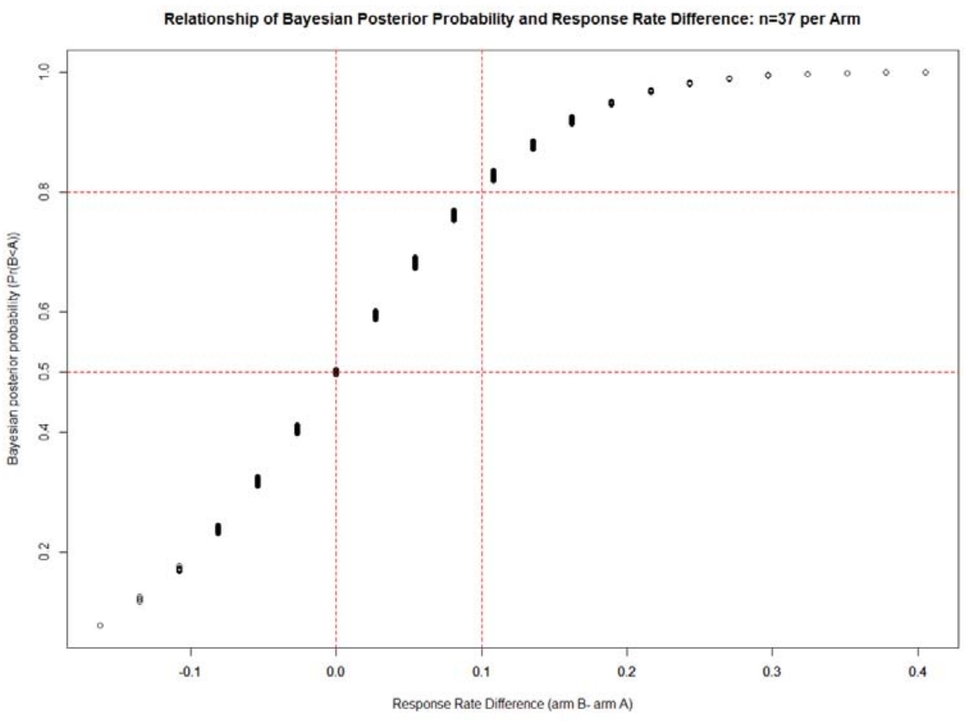

Figure S11B:

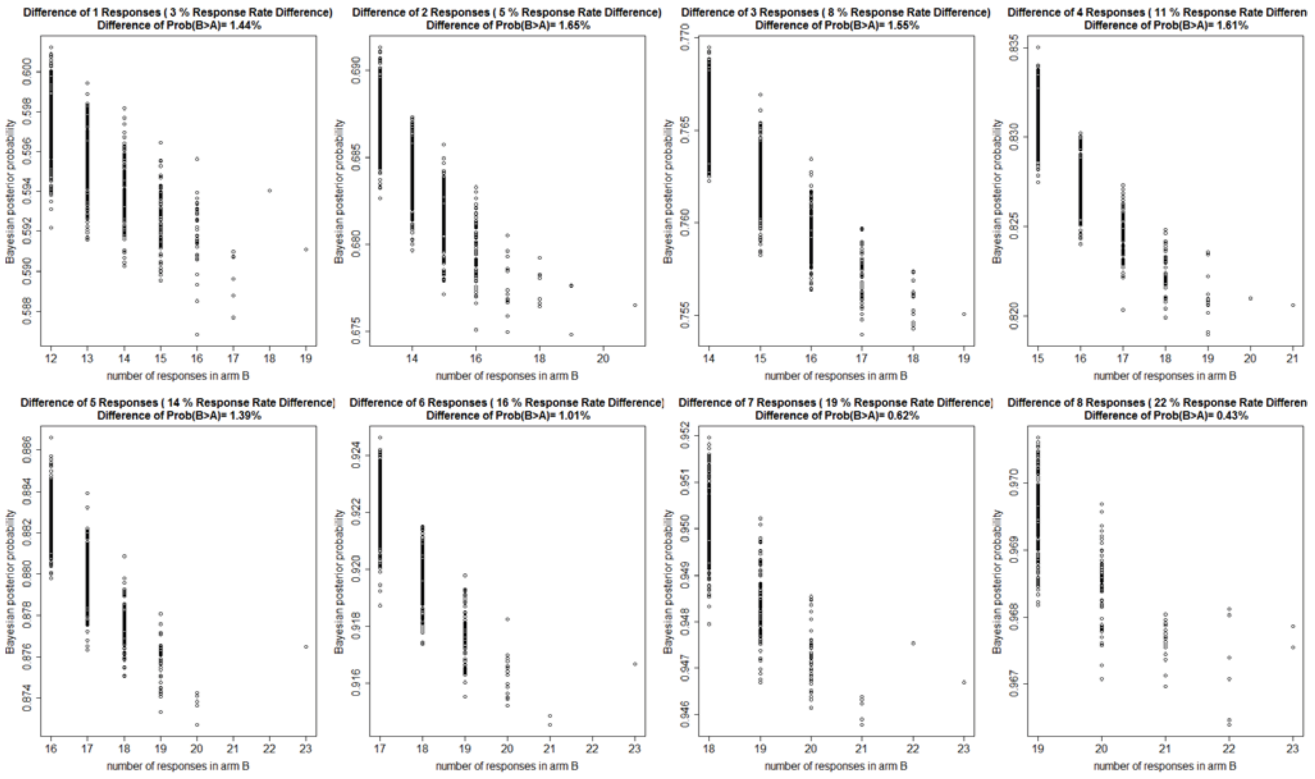

Simulation Study 3: Delta Effect on Local Power and Type I Error.

Since the delta will be implemented only in the case of both arms passing the 2nd stage, we evaluate how the delta affects power and type I error in this subgroup. We use the comparison of 40% versus 20% response rate for illustration (null hypothesis: a 20% response rate in both arms; alternative hypothesis: 40% in arm B and 20% in arm A). Two additional comparisons are also included (45% vs. 30% (15 %difference) and 15% vs. 5% (10% difference)).

Type I error: In the null hypothesis (20% response rate in both arms), the proportion of both arms passing the 2nd stage is 0.93% (Table S1). Half of them (0.47%) misclassify arm B as winner at the delta value of 0.5 (i.e., misclassification rate=50%). As the delta value increases to 0.8 and 0.9, 0.01% or less are claiming arm B as winner.

Power: In the alternative hypothesis (40% in arm B and 20% in arm A, a 20% difference of response rate), there are 9% for both arms passing the 2nd stage. Among them, the power to claim arm B as winner is 8%, 4%, and 2%, for δ=0.5, 0.8, and 0.9, respectively (a 2 fold reduction of power from δ= 0.5 to 0.8, and a 2 fold reduction of power from δ=0.8 to 0.9; Table S1). In the 15% difference of response rate, power reduction is 2-3 folds from δ =0.5 to 0.8.

The additional analyses (45% vs. 30% in Table S2 and 15% vs. 5% in Table S3) show a similar pattern of (1) 50% misclassification rate at δ=0.5 and (2) 3-5 fold power reduction from δ =0.5 to 0.8 for 10%-15% difference of response rate.

In summary, when both arms pass the 2nd stage, a δ of 0.5 will cause to randomly select a winner (misclassification rate=50%). For δ increasing to 0.8 or higher, it will decrease considerably type I error (at least 10 folds), but reduce moderately the power (2-5 folds) to detect a 10%-20% difference of response rate. For this reason, a δ of 0.8 is chosen to determine the winner.

Table S1:
Comparison of 40% vs. 20% response rate

Response rate				Probability of arm B as winner				
			Probability of both	δ				
Arm A	Arm B	difference	arms passing 2nd stage	50%	60%	70%	80%	90%
0.2	0.2	0%	0.0093	0.0047 (50%*)	0.0016	0.0006	0.0001	<0.0001
0.2	0.4	20%	0.0854	0.0762	0.063	0.0523	0.0409	0.0202
0.2	0.35	15%	0.073	0.0588	0.0427	0.0314	0.0213	0.008
0.25	0.4		0.2577	0.2148	0.1739	0.1399	0.1078	0.0513

*: misclassification rate=probability of arm B as winner at δ=50% / probability of both arms passing 2nd stage

Table S2: Comparison of 45% vs. 30% response rate				Probability of arm B as winner				
Response rate		difference	Probability of both arms passing 2nd stage	δ				
Arm A	Arm B			50%	60%	70%	80%	90%
0.3	0.3	0%	0.0185	0.0093 (50%*)	0.0032	0.0015	0.0005	0.0001
0.3	0.45	15%	0.1094	0.0855	0.061	0.0457	0.032	0.0122
0.3	0.4	10%	0.0797	0.0543	0.0322	0.0212	0.0128	0.0034
0.35	0.45		0.267	0.1897	0.132	0.0958	0.066	0.0248
*: misclassification rate=probability of arm B as winner at δ=50% / probability of both arms passing 2nd stage								

Table S3: Comparison of 15% vs. 5% response rate				Probability of arm B as winner				
Response rate		difference	Probability of both arms passing 2nd stage	δ				
Arm A	Arm B			50%	60%	70%	80%	90%
0.05	0.05	0%	0.0107	0.0052 (50%*)	0.0025	0.0007	0.0001	0
0.05	0.15	10%	0.0819	0.0662	0.0582	0.0402	0.0248	0.0069
0.05	0.125	7.25%	0.0682	0.051	0.0413	0.0244	0.013	0.0026
0.075	0.15		0.22	0.165	0.1431	0.0972	0.0582	0.0147
*: misclassification rate=probability of arm B as winner at δ=50% / probability of both arms passing 2nd stage								

Simulation Study 4: Comparison of 45 % Versus 30 % Response Rate

Sample Size Calculation

From historical data, we will consider 30% response rate as not warranting further study. We will use 45% response rate as a promising result to pursue further study. In other words, we are interested in at least 15% (45% vs. 30%) improvement in treatment efficacy for arms B versus A. For each arm, using a Simon Mini-Max two-stage design with 15% type I error rate and 20% type II error rate, 31 patients will be enrolled in the first stage of the trial. If 9 or fewer patients respond, the treatment will be stopped. If 10 or more patients show a response, 7 additional patients (a total of 38 patients per group) will be enrolled. If the total number responding is 14 or less, we will conclude that the treatment is not effective. If both arms fail at the first or second stage, the trial will stop. No winner will be claimed. The sample size will be 62 if both arms fail at the first stage and 69 if only one arm fails at the first stage. If only one arm pass the second stage, the arm will be the winner. If both arms pass the second stage, we will use the posterior probability, $Pr(B > A)$, (probability of the response rate in arm B higher than in arm A) to select the winner. A non-informative prior of beta distribution with parameters of a=1 and b=1 in both arms will be used to calculate the posterior probability. Arm B will be claimed as the winner if $Pr(B > A) > \delta = 0.8$.

Operating Characteristics

The operating characteristics of the design is evaluated by simulation (100000 times) using R software (www.r-project.org) with "clinfun" package. In particular, we are interested in the probability of (correctly) selecting an arm as superior to the other arm if it is truly superior, and conversely, the probability of (incorrectly) selecting an arm that is no better than the other arm.

Power Analysis

Power: Assuming that the true probabilities of response in arms B and A are 45% and 30%, respectively (scenario 1: 15% difference of response rate), the overall probability (power) of correctly choosing arm B as superior is 72% on the basis of superiority shown at the end of the trial. The probability of stopping arm A early and declaring arm B superior at the end of the trial is 69%. There are 11% of both arms passing the second stage with 3% claiming arm B as the winner by the Bayesian posterior probability. In a 10% difference of response rate, the overall power is 52% and 60% for the comparison of arms B and A with 40% versus 30% (scenario 2) and 45% versus 35% (scenario 3), respectively. Proportion of both arms passing the 2nd stage is 8% in scenario 2 (scenario 3: 27%), with 1% (scenario 3: 7%) claiming arm B as the winner by the Bayesian posterior probability.

Type I error

Type I error: In the null hypothesis of a 30% response rate in both arms, there are 12% misclassifying arm B as winner (i.e., 12% type I error). Among them, only 2% has both arms passing the 2nd stage, and less than 0.05% misclassify arm B as winner.

Summary

Summary: With δ=0.8, the design has a 72% power to detect a 15% difference of response rate. The power decreases to a range of 52-60% to differentiate a 10% difference of response rate. The type I error is controlled at 12% when both arms have a 30% response rate.

Tables of Power Anlaysis

Scenario 1: Arm B=0.45 versus Arm A=0.3 (Overall power of Arm B= 72%)

	B.fail.stage1	B.fail.stage2	B.pass
A.fail.stage1	0.03	0.08	0.43
A.fail.stage2	0.02	0.05	0.26
A.pass	0.01	0.02	0.11

Both arms passing the 2nd stage: 11%. Among them, Arm B claims 3.2% as winner

Overall power of Arm B= 72%

Scenario 2: Arm B=0.4 versus Arm A=0.3 (Overall power of Arm B= 52%)

	B.fail.stage1	B.fail.stage2	B.pass
A.fail.stage1	0.08	0.15	0.32
A.fail.stage2	0.05	0.09	0.19
A.pass	0.02	0.04	0.08

Both arms passing the 2nd stage: 8%. Among them, Arm B claims 1.28% as winner

Overall power of Arm B= 52%

Scenario 3: Arm B=0.45 versus Arm A=0.35 (Overall power of Arm B= 60%)

	B.fail.stage1	B.fail.stage2	B.pass
A.fail.stage1	0.02	0.05	0.25
A.fail.stage2	0.02	0.05	0.28
A.pass	0.02	0.05	0.27

Both arms passing the 2nd stage: 27%. Among them, Arm B claims 6.6% as winner

Overall power of Arm B= 60%

Scenario 4: Arm B=0.3 versus Arm A=0.3 (Type I error= 11.94%)

	B.fail.stage1	B.fail.stage2	B.pass
A.fail.stage1	0.29	0.17	0.07
A.fail.stage2	0.17	0.1	0.04
A.pass	0.08	0.04	0.02

Both arms passing the 2nd stage: 2%. Among them, Arm B claims 0.05% as winner

Type I error= 11.94%

Simulation Study 5: Comparison of 15 % Versus 5 % Response Rate

Sample Size Calculation

From historical data, we will consider 5% response rate as not warranting further study. We will use 15% response rate as a promising result to pursue further study. In other words, we are interested in at least 10% (15% vs. 5%) improvement in treatment efficacy for arms B versus A. For each arm, using a Simon Mini-Max two-stage design with 15% type I error rate and 20% type II error rate, 20 patients will be enrolled in the first stage of the trial. If 0 or fewer patients respond, the treatment will be stopped. If 1 or more patients show a response, 16 additional patients (a total of 36 patients per group) will be enrolled. If the total number responding is 3 or less, we will conclude that the treatment is not effective. If both arms fail at the first or second stage, the trial will stop. No winner will be claimed. The sample size will be 40 if both arms fail at the first stage and 56 if only one arm fails at the first stage. If only one arm pass the second stage, the arm will be the winner. If both arms pass the second stage, we will use the posterior probability, $Pr(B > A)$, (probability of the response rate in arm B higher than in arm A) to select the winner. A non-informative prior of beta distribution with parameters of a=1 and b=1 in both arms will be used to calculate the posterior probability. Arm B will be claimed as the winner if $Pr(B > A) > \delta = 0.8$.

Operating Characteristics

The operating characteristics of the design is evaluated by simulation (100000 times) using R software (www.r-project.org) with "clinfun" package. In particular, we are interested in the probability of (correctly) selecting an arm as superior to the other arm if it is truly superior, and conversely, the probability of (incorrectly) selecting an arm that is no better than the other arm.

Power Analysis

Power: Assuming that the true probabilities of response in arms B and A are 15% and 5%, respectively (scenario 1: 10% difference of response rate), the overall probability (power) of correctly choosing arm B as superior is 75% on the basis of superiority shown at the end of the trial. The probability of stopping arm A early and declaring arm B superior at the end of the trial is 72%. There are 8% of both arms passing the second stage with 2% claiming arm B as the winner by the Bayesian posterior probability. In a 8% difference of response rate, the overall power is 61% and 64% for the comparison of arms B and A with 12% versus 5% (scenario 2) and 15% versus 8% (scenario 3), respectively. Proportion of both arms passing the 2nd stage is 7% in scenario 2 (scenario 3: 22%), with 1% (scenario 3: 6%) claiming arm B as the winner by the Bayesian posterior probability.

Type I error

Type I error: In the null hypothesis of a 5% response rate in both arms, there are 9% misclassifying arm B as winner (i.e., 9% type I error). Among them, only 1% has both arms passing the 2nd stage, and less than 0.01% misclassify arm B as winner.

Summary

Summary: With δ=0.8, the design has a 75% power to detect a 10% difference of response rate. The power decreases to a range of 61-64% to differentiate a 8% difference of response rate. The type I error is controlled at 9% when both arms have a 5% response rate.

Tables of Power Anlaysis

Scenario 1: Arm B=0.15 versus Arm A=0.05 (Overall power of Arm B= 75%)

	B.fail.stage1	B.fail.stage2	B.pass
A.fail.stage1	0.01	0.06	0.29
A.fail.stage2	0.02	0.09	0.43
A.pass	0	0.02	0.08

Both arms passing the 2nd stage: 8%. Among them, Arm B claims 2.48% as winner

Overall power of Arm B= 75%

Scenario 2: Arm B=0.125 versus Arm A=0.05 (Overall power of Arm B= 61%)

	B.fail.stage1	B.fail.stage2	B.pass
A.fail.stage1	0.03	0.09	0.24
A.fail.stage2	0.04	0.14	0.36
A.pass	0.01	0.03	0.07

Both arms passing the 2nd stage: 7%. Among them, Arm B claims 1.3% as winner

Overall power of Arm B= 61%

Scenario 3: Arm B=0.15 versus Arm A=0.075 (Overall power of Arm B= 64%)

	B.fail.stage1	B.fail.stage2	B.pass
A.fail.stage1	0.01	0.03	0.17
A.fail.stage2	0.02	0.08	0.41
A.pass	0.01	0.04	0.22

Both arms passing the 2nd stage: 22%. Among them, Arm B claims 5.82% as winner

Overall power of Arm B= 64%

Scenario 4: Arm B=0.05 versus Arm A=0.05 (Type I error= 9%)

	B.fail.stage1	B.fail.stage2	B.pass
A.fail.stage1	0.13	0.19	0.04
A.fail.stage2	0.19	0.29	0.05
A.pass	0.04	0.06	0.01

Both arms passing the 2nd stage: 1%. Among them, Arm B claims 0.01% as winner

Type I error= 9%

Simulation Study 6: Fleming Single Stage Design (A prototype)

Hypothesis: Comparison of 40% versus 20% Response Rate

Sample Size Calculation

From historical data, we will consider 20% response rate as not warranting further study. We will use 40% response rate as a promising result to pursue further study. In other words, we are interested in at least 20% (40% vs. 20%) improvement in treatment efficacy for arms B versus A. For each arm, using the Fleming single stage design with 10% type I error rate and 10% type II error rate, 36 patients will be enrolled in the trial. If 11 patients or more respond, the treatment will be considered competitive. If both arms fail, the trial will stop. No winner will be claimed. The sample size will be 36 patients per arm. If only one arm is competitive (i.e., number of responses >10), the arm will be the winner. If both arms are competitive, we will use the posterior probability, Pr(B>A), (probability of the response rate in arm B higher than in arm A) to select the winner. A non-informative prior of beta distribution with parameters of a=1 and b=1 in both arms will be used to calculate the posterior probability. Arm B will be claimed as the winner if Pr(B>A)>delta=0.8.

Operating Characteristics

The operating characteristics of the design is evaluated by simulation (10000 times) using R software (www.r-project.org) with "clinfun" package. In particular, we are interested in the probability of (correctly) selecting an arm as superior to the other arm if it is truly superior, and conversely, the probability of (incorrectly) selecting an arm that is no better than the other arm.

Power Analysis

Power: Assuming that the true probabilities of response in arms B and A are 40% and 20%, respectively (scenario 1: 20% difference of response rate), the overall probability (power) of correctly choosing arm B as superior is 86% on the basis of superiority shown at the end of the trial. The probability of stopping arm A early and declaring arm B superior at the end of the trial is 82%. There are 8% of both arms being competitive with 4% claiming arm B as the winner by the Bayesian posterior probability. In a 15% difference of response rate, the overall power is 71% and 75% for the comparison of arms B and A with 35% versus 20% (scenario 2) and 40% versus 25% (scenario 3), respectively. Proportion of both arms being competitive is 7% in scenario 2 (scenario 3: 25%), with 2% (scenario 3: 10%) claiming arm B as the winner by the Bayesian posterior probability.

Type I error

Type I error: In the null hypothesis of a 20% response rate in both arms, there are 8% misclassifying arm B as winner (i.e., 8% type I error). Among them, only 1% has both arms being competitive, and 0.01% misclassify arm B as winner.

Summary

Summary: With delta=0.8, the design has a 86% power to detect a 20% difference of response rate. The power decreases to a range of 71-75% to differentiate a 15% difference of response rate. The type I error is controlled at 8% when both arms have a 20% response rate.

Table of Power Analysis

```
-----------------------------------------------
Scenario 1: Arm B=0.4 versus Arm A=0.2 (Overall power of Arm B= 86%)



|       |B.fail |B.pass |
|:------|:------|:------|
|A.fail |0.09   |0.82   |
|A.pass |0.01   |0.08   |

 Both arms pass: 8%. Among them,  Arm B claims 3.53% as winner

 Overall power of Arm B= 86%

-----------------------------------------------
Scenario 2: Arm B=0.35 versus Arm A=0.2 (Overall power of Arm B= 71%)



|       |B.fail |B.pass |
|:------|:------|:------|
|A.fail |0.22   |0.7    |
|A.pass |0.02   |0.07   |

 Both arms pass: 7%. Among them,  Arm B claims 1.76% as winner

 Overall power of Arm B= 71%

-----------------------------------------------
```

```
Scenario 3: Arm B=0.4 versus Arm A=0.25 (Overall power of Arm B= 75%)



|       |B.fail |B.pass |
|:------|:------|:------|
|A.fail |0.07   |0.65   |
|A.pass |0.03   |0.25   |

 Both arms pass: 25%. Among them,  Arm B claims 9.91% as winner

 Overall power of Arm B= 75%

----------------------------------------------
Scenario 4: Arm B=0.2 versus Arm A=0.2 (Type I error= 8%)



|       |B.fail |B.pass |
|:------|:------|:------|
|A.fail |0.82   |0.08   |
|A.pass |0.08   |0.01   |

 Both arms pass: 1%. Among them,  Arm B claims 0.01% as winner

 Type I error= 8%
```

Comparison 1: Comparison of the Delta, δ, in Bayesian Pick-the-Winner Design and the Difference of Response Rates, d, in the Sargent And Goldberg's Method

Both serve the same functionality as a threshold to determine the winner.

Sargent and Goldberg's method:

H0: $\pi_a = \pi_b$ v.s. H1: $\pi_a \neq \pi_b$ (π_a and π_b are true response rate) (two-sided test)

T statistics= $\frac{|p_a - p_b|}{\hat{\sigma}} \sim Z_{\alpha/2}$,

where

$\hat{\sigma} = [((p_a(1-p_a) + p_b(1-p_b))/n]^{1/2}$,

p_a and p_b are the observed response rate.

d= $\hat{\sigma} * Z_{\alpha/2}$

$\delta = \pi_a - \pi_b$

Claim that H0 is rejected if $|p_a - p_b|$ > d

So 'd' serves as a cutoff for difference of observed response rates.

$\beta = \Phi\left(Z_{\alpha/2} - \delta/\hat{\sigma}\right) - \Phi\left(-Z_{\alpha/2} - \delta/\hat{\sigma}\right)$

Power=$P(p_A > p_B + d) + \rho P(p_B + d > p_A > p_B - d)$= $1 - \Phi\left(Z_{\alpha/2} - \delta/\hat{\sigma}\right) + \rho\beta$

For one-sided test with ρ=0,

d= $\widehat{\sigma} * Z_{\alpha}$

Power = $1 - \Phi(Z_{\alpha} - \delta/\widehat{\sigma})$

When $Z_{\alpha} = 0$ (i.e., α=0.5), d will be 0. The decision rule becomes a random choice. This is equivalent to δ=0.5 in Bayesian pick-the-winner design.

With $\pi_a = 40\%, \pi_b = 20\%$, n=37 per arm, and d=0.1,

$\widehat{\sigma}$= sqrt((0.4*0.6+0.2*0.8)/37)= 0.103975

$Z_{\alpha} = d/\widehat{\sigma}$= 0.9617692.
Thus, α= 0.1680828~=17% (type I error).

Power= $1 - \Phi(Z_{\alpha} - \delta/\widehat{\sigma}) = 1 - \Phi(0.9617692 - 0.2/0.103975)$=83%.

For the Bayesian pick-the-winner design, with the comparison of 40% vs. 20% response rate and δ=0.8, the power is 86%.

In addition, there exists a positive correlation between the Bayesian posterior probability and the response rate difference. As shown in the Supplementary Simulation Study 2, we find an increasing pattern of Bayesian posterior probability as the difference of response rate increases, but it is not a one-to-one relationship.

Summary: Both δ and d function as a threshold to determine the winner. Specifically, δ is used as a cutoff in the Bayesian posterior probability while d works as a cutoff for the difference of observed response rates. Although they are in a different metric, they are highly correlated.

R package 'BayesianPickWinner**":**

1. Install the package:
   devtools::install_github("dungtsa/BayesianPickWinner",force = TRUE)
2. Load the library: require(BayesianPickWinner)
3. Run the Shiny application: bayes.pick.winner()
4. A web browser will be started for graphical user interface (GUI) to allow users to
   a. Explore various settings of response rates for power analysis (see snapshots below)
   b. Calculate the Bayesian posterior probability when the trial reaches to the end of the 2nd stage to determine the winner between two competitive arms.

Snapshot of shiny app: initial

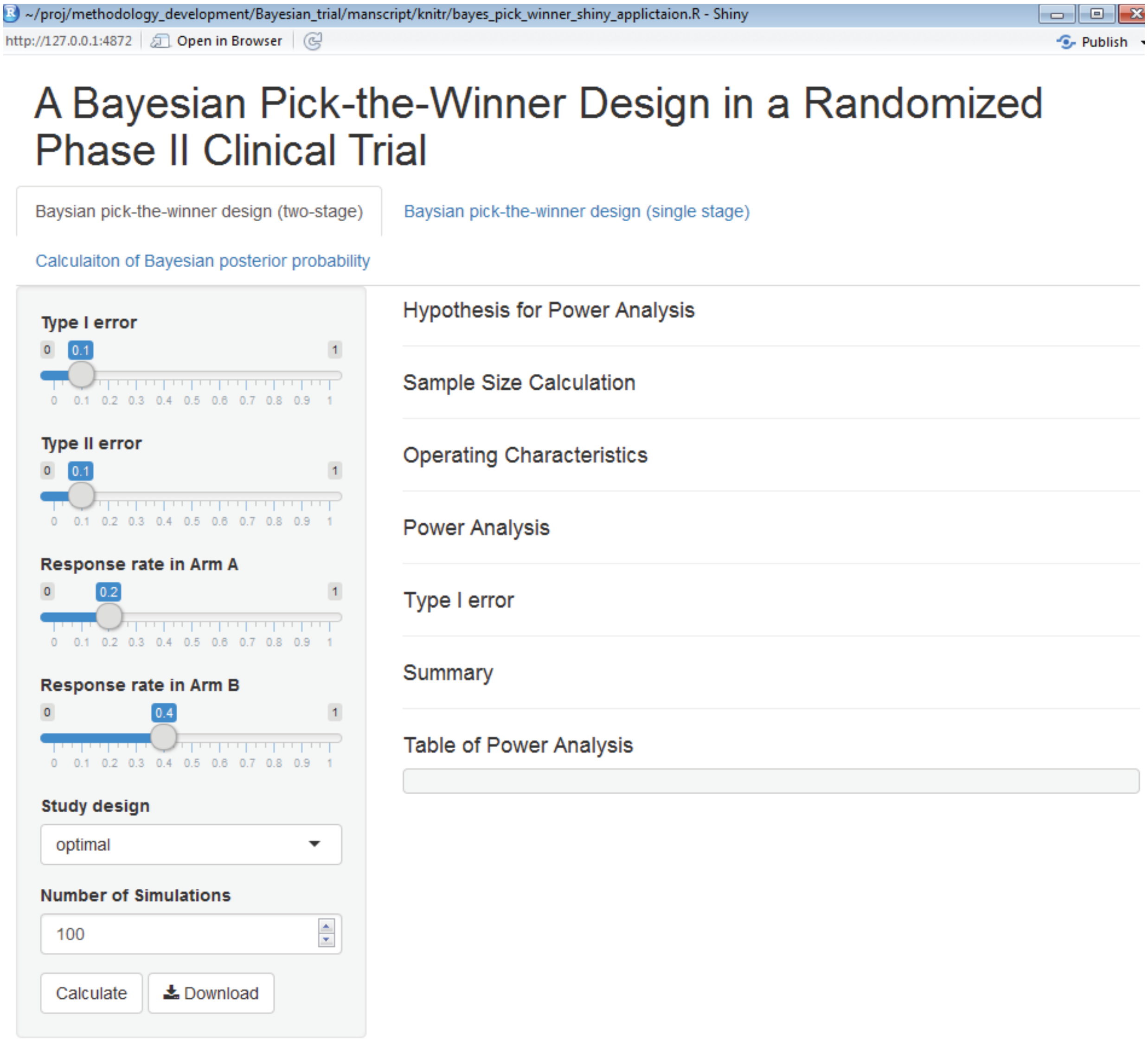

Snapshot of shiny app: output

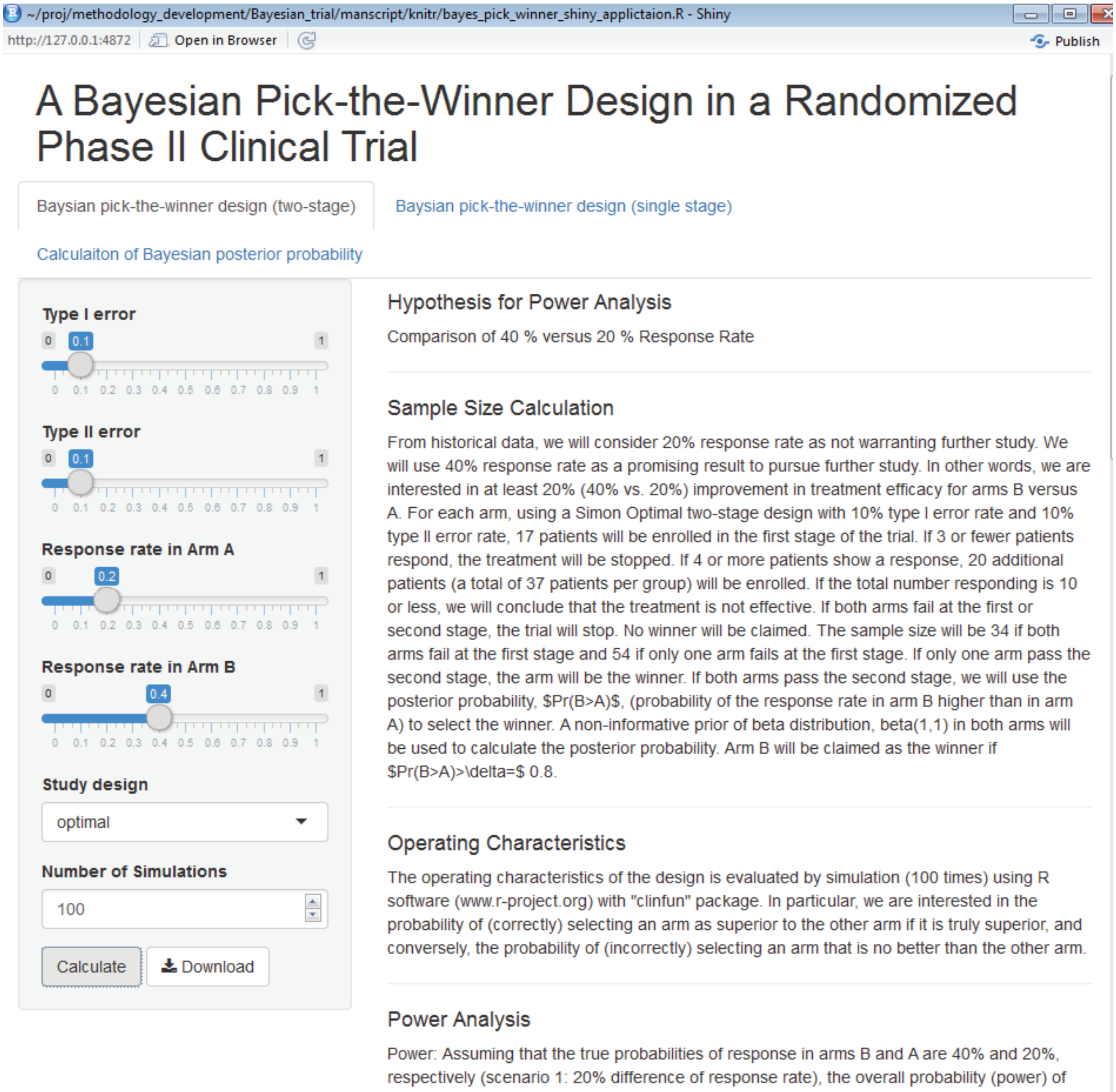

Snapshot of shiny app: output in Word format (through "download" button)

A Bayesian Pick-the-Winner design in a randomized phase II clinical trial

Chen et.al

==

Hypothesis for Power Analysis

Comparison of 40 % versus 20 % Response Rate

Sample Size Calculation

From historical data, we will consider 20% response rate as not warranting further study. We will use 40% response rate as a promising result to pursue further study. In other words, we are interested in at least 20% (40% vs. 20%) improvement in treatment efficacy for arms B versus A. For each arm, using a Simon Optimal two-stage design with 10% type I error rate and 10% type II error rate, 17 patients will be enrolled in the first stage of the trial. If 3 or fewer patients respond, the treatment will be stopped. If 4 or more patients show a response, 20 additional patients (a total of 37 patients per group) will be enrolled. If the total number responding is 10 or less, we will conclude that the treatment is not effective. If both arms fail at the first or second stage, the trial will stop. No winner will be claimed. The sample size will be 34 if both arms fail at the first stage and 54 if only one arm fails at the first stage. If only one arm pass the second stage, the arm will be the winner. If both arms pass the second stage, we will use the posterior probability, $Pr(B > A)$, (probability of the response rate in arm B higher than in arm A) to select the winner. A non-informative prior of beta distribution, beta(1,1) in both arms will be used to calculate the posterior probability. Arm B will be claimed as the winner if $Pr(B > A) > \delta = 0.8$.

Operating Characteristics

The operating characteristics of the design is evaluated by simulation (100 times) using R software (www.r-project.org) with "clinfun" package. In particular, we are interested in the probability of (correctly) selecting an arm as superior to the other arm if it is truly superior, and conversely, the probability of (incorrectly) selecting an arm that is no better than the other arm.

Power Analysis

Power: Assuming that the true probabilities of response in arms B and A are 40% and 20%, respectively (scenario 1: 20% difference of response rate), the overall probability (power) of correctly choosing arm B as superior is 85% on the basis of superiority shown at the end of the trial. The probability of stopping arm A early and declaring arm B superior at the end of the trial is 82%. There are 7% of both arms passing the second stage with 3% claiming arm B as the winner by the Bayesian posterior probability. In a 15% difference of response rate, the overall power is 70% and 73% for the comparison of arms B and A with 35% versus 20% (scenario 2) and 40% versus 25% (scenario 3), respectively. Proportion of both arms passing the 2nd stage is 7% in scenario 2 (scenario 3: 20%), with 4% (scenario 3: 9%) claiming arm B as the winner by the Bayesian posterior probability.

Type I error

Type I error: In the null hypothesis of a 20% response rate in both arms, there are 7% misclassifying arm B as winner (i.e., 7% type I error). Among them, only 0% has both arms passing the 2nd stage, and less than 0% misclassify arm B as winner.

Summary

Summary: With δ=0.8, the design has a 85% power to detect a 20% difference of response rate. The power decreases to a range of 70-73% to differentiate a 15% difference of response rate. The type I error is controlled at 7% when both arms have a 20% response rate.

Tables of Power Anlaysis

Scenario 1: Arm B=0.4 versus Arm A=0.2 (Overall power of Arm B= 85%)

	B.fail.stage1	B.fail.stage2	B.pass
A.fail.stage1	0.05	0.01	0.62
A.fail.stage2	0.01	0.03	0.2
A.pass	0	0.01	0.07

Both arms passing the 2nd stage: 7%. Among them, Arm B claims 3% as winner

Overall power of Arm B= 85%

Scenario 2: Arm B=0.35 versus Arm A=0.2 (Overall power of Arm B= 70%)

	B.fail.stage1	B.fail.stage2	B.pass
A.fail.stage1	0.11	0.08	0.42
A.fail.stage2	0.01	0.05	0.24
